# Identification of pathogenic genes and upstream regulators in age-related macular degeneration

**DOI:** 10.1186/s12886-017-0498-z

**Published:** 2017-06-26

**Authors:** Bin Zhao, Mengya Wang, Jing Xu, Min Li, Yuhui Yu

**Affiliations:** 1 Department of Ophthlmology, Affiliated Hospital of Taishan Medical College, No.706 Taishan street, Taian, 271000 China; 2 Department of Ophthlmology, Peoples Hospital of Feicheng, No. 108 Xincheng Road, Feicheng, 271699 China; 3 Department of Ophthlmology, The First Peoples Hospital of Jining, Shandong, No.6 Jiankang Road, Jining, 272011 China; 4 Department of Ophthlmology, The Third Peoples Hospital of Xintai, No.127 Cuyang street, Taian, 271212 China

**Keywords:** Age-related macular degeneration, Transcription factors, Differentially expressed genes, Integrated analysis

## Abstract

**Background:**

Age-related macular degeneration (AMD) is the leading cause of irreversible blindness in older individuals. Our study aims to identify the key genes and upstream regulators in AMD.

**Methods:**

To screen pathogenic genes of AMD, an integrated analysis was performed by using the microarray datasets in AMD derived from the Gene Expression Omnibus (GEO) database. The functional annotation and potential pathways of differentially expressed genes (DEGs) were further discovered by Gene Ontology (GO) and Kyoto Encyclopedia of Genes and Genomes (KEGG) enrichment analysis. We constructed the AMD-specific transcriptional regulatory network to find the crucial transcriptional factors (TFs) which target the DEGs in AMD. Quantitative real time polymerase chain reaction (qRT-PCR) was performed to verify the DEGs and TFs obtained by integrated analysis.

**Results:**

From two GEO datasets obtained, we identified 1280 DEGs (730 up-regulated and 550 down-regulated genes) between AMD and normal control (NC). After KEGG analysis, steroid biosynthesis is a significantly enriched pathway for DEGs. The expression of 8 genes (TNC, GRP, TRAF6, ADAMTS5, GPX3, FAP, DHCR7 and FDFT1) was detected. Except for TNC and GPX3, the other 6 genes in qRT-PCR played the same pattern with that in our integrated analysis.

**Conclusions:**

The dysregulation of these eight genes may involve with the process of AMD. Two crucial transcription factors (c-rel and myogenin) were concluded to play a role in AMD. Especially, myogenin was associated with AMD by regulating TNC, GRP and FAP. Our finding can contribute to developing new potential biomarkers, revealing the underlying pathogenesis, and further raising new therapeutic targets for AMD.

**Electronic supplementary material:**

The online version of this article (doi:10.1186/s12886-017-0498-z) contains supplementary material, which is available to authorized users.

## Background

Age-related macular degeneration (AMD) is the leading cause of irreversible blindness in older individuals [1, 2]. Pathological deposits (drusen) between the Bruch’s membrane and retinal pigment epithelium (RPE) are closely associated with early stage AMD [3]. Late AMD can be classified into the dry form and the wet form [4]. Dry AMD is mainly caused by atrophy of RPE. Wet AMD is characterized by choroidal neovascularization (CNV), subretinal haemorrhage, photoreceptor loss, retinal detachment and visual loss [5, 6].

Age is the major risk factor associated with AMD, but beyond that, vascular endothelial growth factor (VEGF), oxidative stress, inflammation, complement system are associated with risk of AMD [7]. Despite many efforts for AMD research in recent decades, effective treatments remain absent [8, 9].

With advances in various high-throughput technologies, amounts of key genes which are associated with the process of disease were identified by microarray [10–12]. Using multiple microarray datasets, integrated analysis could identified differentially expressed genes (DEGs) with more accuracy, and increased the statistical power compared with a single microarray study. Exploring the upstream transcription factors (TFs) mediating abnormal gene expression in disease status can help to understand pathophysiological changes in complex diseases.

In this study, we performed integrated analysis of multiple microarray datasets to identify DEGs between AMD and normal control (NC) groups, which may be used as potential diagnostic biomarkers for AMD. In addition, we identified several crucial TFs which target the DEGs in AMD. The aim of this study is to better characterize the molecular events and pathways of AMD and to raise new strategies of treatment for AMD.

## Methods

### Eligible gene expression profiles of AMD

We selected gene expression datasets of AMD on the Gene Expression Omnibus database (GEO, http://www.ncbi.nlm.nih.gov/geo) which is the largest database of high-throughput gene expression data. Selected datasets should be whole-genome expression profile data in AMD patients and NC groups. Datasets with drug stimulation or transfection were excluded.

### Identify DEGs in AMD compared to NC

Background correction was performed for the raw data. Differential expression *p*-values were calculated by two-tailed Student’s t-test, we combined these *p*-values by the inverse normal method. Finally, the DEGs between AMD and NC groups were identified with criterion of *P*-value < 0.05.

### Functional annotation

To reveal the function and the potential pathway of DEGs, Gene Ontology (GO) classification (molecular functions, biological processes and cellular component) and Kyoto Encyclopedia of Genes and Genomes (KEGG) pathway enrichment were performed by using the online software GeneCodis (http://genecodis.cnb.csic.es/analysis).FDR < 0.05 was defined as the criteria of statistical significance.

### AMD-specific protein-protein interaction (PPI) network construction

To further research the biological functions of DEGs, the top 30 DEGs in AMD were used to construct the PPI network by using Biological General Repository for Interaction Datasets (BioGRID) (http://thebiogrid.org/) and Cytoscape. We used nodes and edges to represent the proteins and interactions between two proteins respectively.

### Construction of AMD-specific transcriptional regulatory networks

TFs could regulate gene transcription by binding to specific DNA sequences generally located in the promoter region of genes. Based on the integrated analysis, the corresponding promoters of the top ten up-regulated or down-regulated DEGs were obtained by UCSC Genome Bioinformatics (http://genome.ucsc.edu).TheTFsinvolved in regulating these DEGs were derived from the match tools in TRANSFAC which is a database of transcription factor. The transcriptional regulatory network was constructed by Cytoscape software (http://www.cytoscape.org/).

### qRT-PCR confirmation

Patients with wet AMD in evolutionary stage and sex- and age-matched normal controls were recruited in our study. The exclusion criteria for AMD patients were as follows: (1) Patients with other ocular organic disease; (2) Patients with glaucoma, polypoidal choroidal vasculopathy and other ocular lesions. The NCs with ocular lesions or other serious medical illness were excluded. The detailed characteristics of AMD patients were displayed in Table 1.

**Table 1:** Patient characteristics

	Case 1	Case 2	Case 3
Age (years)	78	66	77
Type	Wet	Wet	Wet
Stage	Evolutionary stage	Evolutionary stage	Evolutionary stage
UCVA	0.6	0.5	0.3
BCVA	1.2	1.4	1.2
CFT(cm)	378	362	412
Fundus performance	Choroidal neovascularization	Choroidal neovascularization	Choroidal neovascularization
FFA	Fluorescein leakage	Fluorescein leakage	Fluorescein leakage
ICGA	Choroidal neovascularization	Choroidal neovascularization	Choroidal neovascularization
OCT	Macular edema and morphological deformation	Macular edema and morphological deformation	Macular edema and morphological deformation

*UCVA* Uncorrected visual acuity, *BCVA* best corrected visual acuity, *CFT* central fovea thickness, *FFA* Fundus fluorescein angiograph, *ICGA* Indo cyanine green angiography, *OCT* Optical Coherence mography

Blood samples from 3 ADM patients (Case 1–3) and 3 NCs were collected and frozen at 80 °C within 2 h after blood withdrawal. After thawing the frozen samples at room temperature, 1 ml of the sample was used to perform RNA isolation with Trizol reagent (Invitrogen, China) according to the manufacturer’s instructions. By using SuperScript® III Reverse Transcriptase (Invitrogen, China), we generated cDNA from 1μgextracted RNA. With Power SYBR® Green PCR Master Mix (Applied Biosystems, USA), we performed quantitative PCR in an ABI 7500 real-time PCR system. Relative gene expression was analyzed using 2^-ΔΔCt^ method. The human 18srRNA was used as endogenous control for mRNA expression in analysis.

## Results

### Differential expression analysis of genes in AMD

**Fig. 1:**
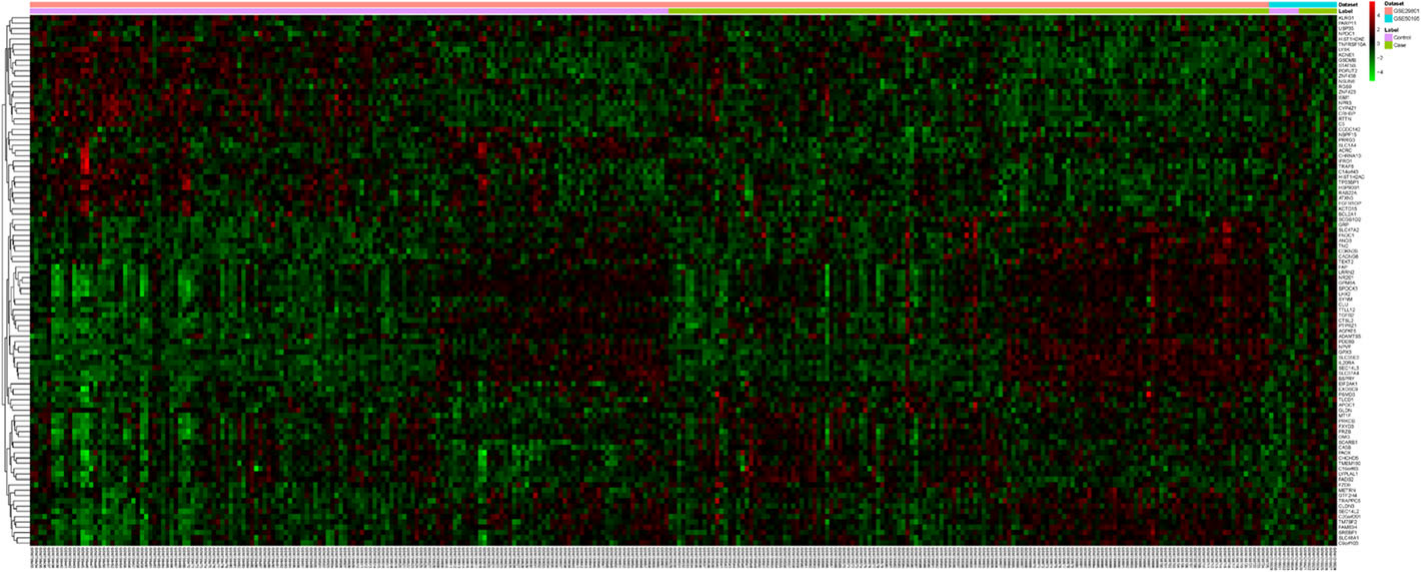
Heat-map image displaying top 100 genes that were significantly up-regulated or down-regulated (*P*-value < 0.05) in AMD compared to normal controls

Two RNA-seq datasets (GSE90889 and GSE67898) of AMD were excluded due to that the data of these two datasets were obtained after treatment with nicotinamide and TGFβ receptor kinase inhibitor, A-83-01, respectively. Two gene expression microarray datasets (GSE50195, GSE29801) without drug stimulation or transfection were enrolled in this study (Table 2). Compared with the normal controls, 1280 DEGs in AMD were obtained with *P* < 0.05, among which, 730 genes were up-regulated and 550 genes were down-regulated. The top 30 most significantly up or down-regulated genes were listed in Table 3.The heat map of top 100 most significantly up or down-regulated genes (*P*-value < 0.05) in AMD was showed in Fig. 1.

**Table 2:** List of mRNA study samples from GEO

GEO accession	Author	Platform	Samples (N:P)	Year
GSE50195	Whitmore SS	GPL17629[HuEx-1_0-st] Affymetrix Human Exon 1.0 ST Array [AltAnalyzprobeset-to-Ensembl mapping]	9:7	2013
GSE29801	Radeke MJ	GPL4133 Agilent-014850 Whole Human Genome Microarray 4x44K G4112F (Feature Number version)	151:142	2012

**Table 3:** Top 30 most significantly up- or down-regulated genes in AMD

GeneId	Symbol	Combined.ES	P.Value	FDR	Regulation
3371	TNC	0.583092249	4.83E-07	0.002942856	Up
341	APOC1	0.56999013	8.86E-07	0.002942856	Up
54,742	LY6K	−0.568356126	9.64E-07	0.002942856	Down
10,647	SCGB1D2	0.564849096	1.05E-06	0.002942856	Up
84,624	FNDC1	0.542691118	2.64E-06	0.005012426	Up
2922	GRP	0.543254919	2.67E-06	0.005012426	Up
11,096	ADAMTS5	0.542022774	2.72E-06	0.005012426	Up
146,802	SLC47A2	0.5296511	4.48E-06	0.006043125	Up
55,652	SLC48A1	0.529070013	4.69E-06	0.006043125	Up
5803	PTPRZ1	0.520669191	6.49E-06	0.007684835	Up
2191	FAP	0.51940808	6.88E-06	0.007684835	Up
7189	TRAF6	−0.517257899	7.61E-06	0.007741325	Down
6720	SREBF1	0.514810439	8.35E-06	0.007779644	Up
7158	TP53BP1	−0.504884978	1.20E-05	0.010594686	Down
64,111	NPVF	0.50028869	1.45E-05	0.011148798	Up
23,336	SYNM	0.501243284	1.47E-05	0.011148798	Up
116,238	TLCD1	0.498263445	1.53E-05	0.011148798	Up
2878	GPX3	0.496829715	1.64E-05	0.011475202	Up
414,328	C9orf103	0.483245526	2.70E-05	0.017394853	Up
79,047	KCTD15	−0.48163493	2.90E-05	0.018035511	Down
199,974	CYP4Z1	−0.47837158	3.37E-05	0.019718857	Down
3475	IFRD1	−0.477678809	3.41E-05	0.019718857	Down
8334	HIST1H2AC	−0.467574449	4.95E-05	0.026761718	Down
84,269	CHCHD5	0.463229503	5.82E-05	0.029154389	Up
949	SCARB1	0.464706572	5.91E-05	0.029154389	Up
7101	NR2E1	0.46403246	6.20E-05	0.029680373	Up
5393	EXOSC9	0.458296775	6.96E-05	0.030872051	Up
10,219	KLRG1	−0.454787798	7.61E-05	0.031906258	Down
23,090	ZNF423	−0.451945937	8.69E-05	0.034135755	Down
57,097	PARP11	−0.450396437	8.96E-05	0.034135755	Down

### Functional annotation

**Fig. 2:**
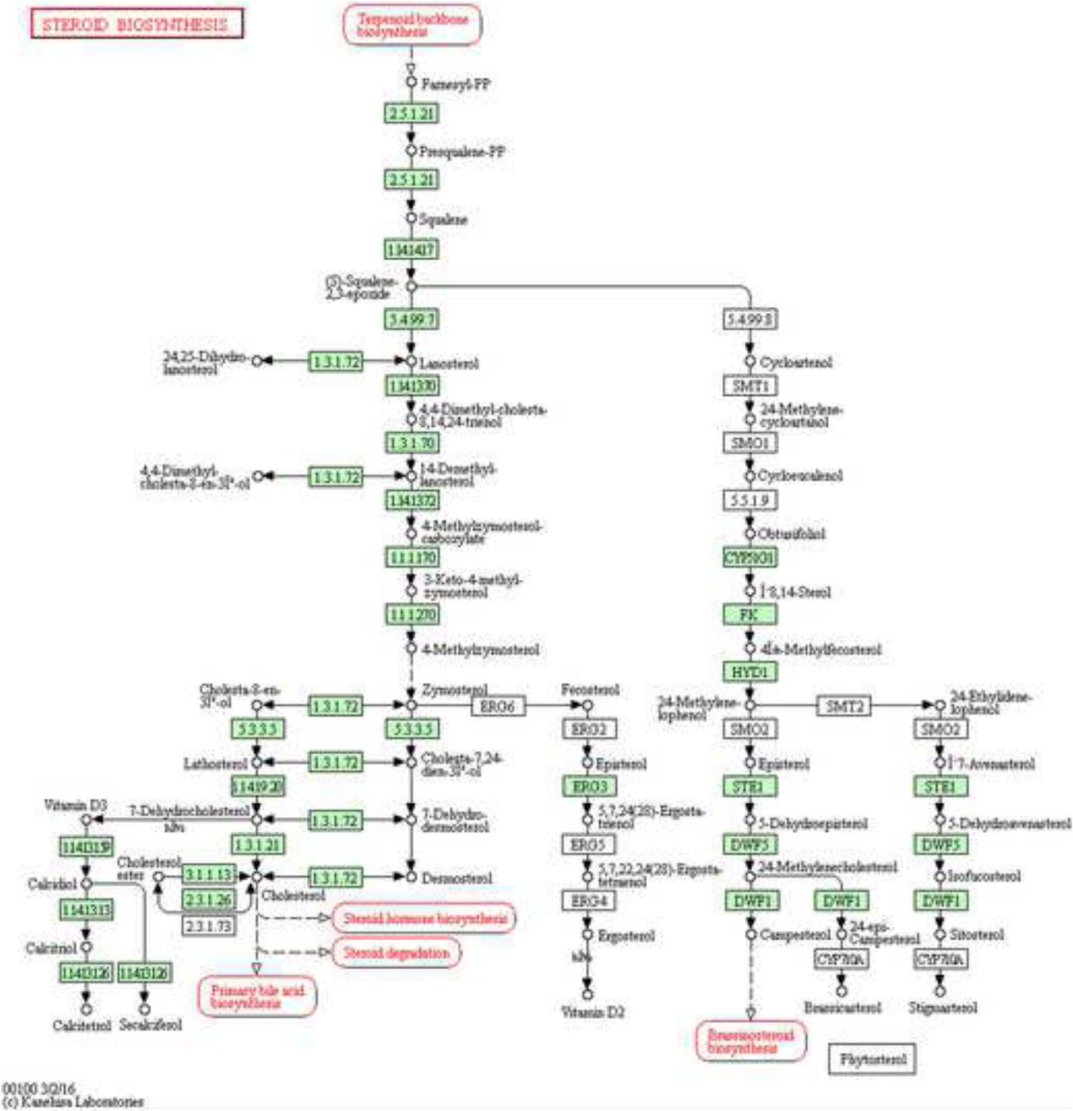
Steroid biosynthesis that enriched in differentially expressed genes of AMD The red rectangles were represented the elements which regulated by the differentially expressed genes that enriched in steroid biosynthesis

As the results of GO enrichment analysis (FDR < 0.05, Additional file 1: Table S1), the DEGs were significantly enriched in regulation of transcription, DNA-dependent (FDR = 4.74E-08), cellular nitrogen compound metabolic process (FDR = 1.25E-06), cholesterol biosynthetic process (FDR = 1.30E-06), membrane (FDR = 1.53E-31), cytoplasm (FDR = 2.17E-31), protein binding (FDR = 3.70E-30) and DNA binding (FDR = 4.60E-17). After the KEGG pathway enrichment analysis (FDR < 0.05, Table 4), several pathways were significantly enriched, including valine, leucine, isoleucine degradation (FDR = 4.19E-08), steroid biosynthesis (FDR = 1.74E-07) and WNT signaling pathway (FDR = 1.02E-05). 8 DEGs (DHCR7, FDFT1, CYP27B1, TM7SF2, SQLE andSOAT2) were significantly enriched in the steroid biosynthesis which are associated with AMD (Table 5 and Fig. 2).

**Table 4:** The top 15 most significantly enriched pathways in AMD

Items	Items_Details	Count	P_value	FDR	Genes
Kegg:00280	Valine, leucine and isoleucine degradation	12	4.19E-08	8.51E-06	ACAT2,ALDH3A2,BCAT1,HMGCS1,BCKDHB,BCKDHA,ALDH9A1,HMGCL,ACADSB,IL4I1,PCCB,ALDH2
Kegg:00100	Steroid biosynthesis	8	1.74E-07	1.77E-05	DHCR7,FDFT1,CYP27B1,TM7SF2,SQLE,SOAT2,DHCR24,EBP
Kegg:04310	Wnt signaling pathway	18	1.02E-05	0.000516	LRP5,LEF1,NLK,CAMK2B,PRKCB,NFATC3,CSNK1E,PRKCG,FZD5,PPP2R5C,SFRP2,FZD9,APC,CTNNBIP1,ROCK1,DKK1,APC2,MMP7
Kegg:00900	Terpenoid backbone biosynthesis	6	9.28E-06	0.000628	ACAT2,HMGCS1,MVK,GGPS1,IDI1,FDPS
Kegg:05322	Systemic lupus erythematosus	13	1.99E-05	0.00081	HIST1H2BM,HIST1H2BJ,HIST1H2BD,C5,HIST1H2BL,HIST1H2BH,CTSG,CD86,HIST1H2AC,HIST3H2A,HIST1H2AE,HIST1H2BB,HIST1H2BO
Kegg:04080	Neuroactive ligand-receptor interaction	24	6.26E-05	0.002118	P2RY2,GABRB1,CHRNA3,ADRB1,GCGR,GABRE,TBXA2R,GRM7,BDKRB2,CALCRL,NMBR,NPY1R,HRH3,SSTR1,GRM5,CTSG,CHRNA10,MC3R,GRIK3,GABRP,RXFP1,TACR3,CRHR1,ADORA1
Kegg:04020	Calcium signaling pathway	18	8.83E-05	0.002242	ADRB1,CAMK2B,TBXA2R,BDKRB2,PRKCB,MYLK,PRKCG,GRM5,ERBB4,ADCY7,ITPKA,PLCZ1,SLC25A4,NOS3,TACR3,PHKG1,RYR1,PDE1A
Kegg:05200	Pathways in cancer	27	7.78E-05	0.002255	FH,LEF1,RARB,PIAS2,FGF1,PRKCB,RUNX1T1,PRKCG,NRAS,TGFB3,CDKN2B,FZD5,TGFB2,EGF,TRAF6,FZD9,APC,GSTP1,HSP90B1,HDAC1,PAX8,MAP2K1,PPARG,STAT5B,CBL,APC2,RALGDS
Kegg:00330	Arginine and proline metabolism	9	0.000145	0.003266	ALDH3A2,ADC,ALDH9A1,ARG1,NOS3,GOT2,AMD1,ALDH4A1,ALDH2
Kegg:04350	TGF-beta signaling pathway	11	0.000207	0.004199	BMP7,COMP,BMPR1B,TGFB3,ID1,CDKN2B,SMURF2,TGFB2,THBS4,ROCK1,ZFYVE9
Kegg:04012	ErbB signaling pathway	11	0.00035	0.006461	CAMK2B,PRKCB,PRKCG,NRAS,EREG,ERBB4,EGF,PAK3,MAP2K1,STAT5B,CBL
Kegg:00310	Lysine degradation	8	0.00039	0.006594	ACAT2,ALDH3A2,PIPOX,WHSC1L1,ALDH9A1,SETD8,GLT25D2,ALDH2
Kegg:04146	Peroxisome	10	0.000466	0.00727	PAOX,PIPOX,SLC27A2,MVK,AGXT,PEX7,HMGCL,NUDT12,PEX10,AMACR
Kegg:04142	Lysosome	13	0.000534	0.007738	MCOLN1,NAGPA,SCARB2,CTSF,CTSL2,ARSA,CTSG,LAMP1,ATP6AP1,SORT1,LAPTM4B,ABCB9,AP3S1
Kegg:01040	Biosynthesis of unsaturated fatty acids	5	0.000836	0.009981	PTPLB,FADS2,SCD,ACOT1,FADS1

**Table 5:** Differentially expressed genes enriched in steroid biosynthesis

Symbol	Gene annotation	Enzyme	Up down
DHCR7	delta 7-dehydrocholesterol reductase	EC:1.3.1.21	Up
FDFT1	Farnesyl-DiphosphateFarnesyltransferase 1.	EC:2.5.1.21	Up
CYP27B1	cytochrome P450 family 27 subfamily B member 1	EC:1.14.13.13	Up
TM7SF2	transmembrane 7 superfamily member 2	EC:1.3.1.70	Up
SQLE	squaleneepoxidase	EC:1.14.14.17	Up
SOAT2	sterol O-acyltransferase 2	EC:2.3.1.26	Down

### AMD-specific protein-protein interaction (PPI) network

**Fig. 3:**
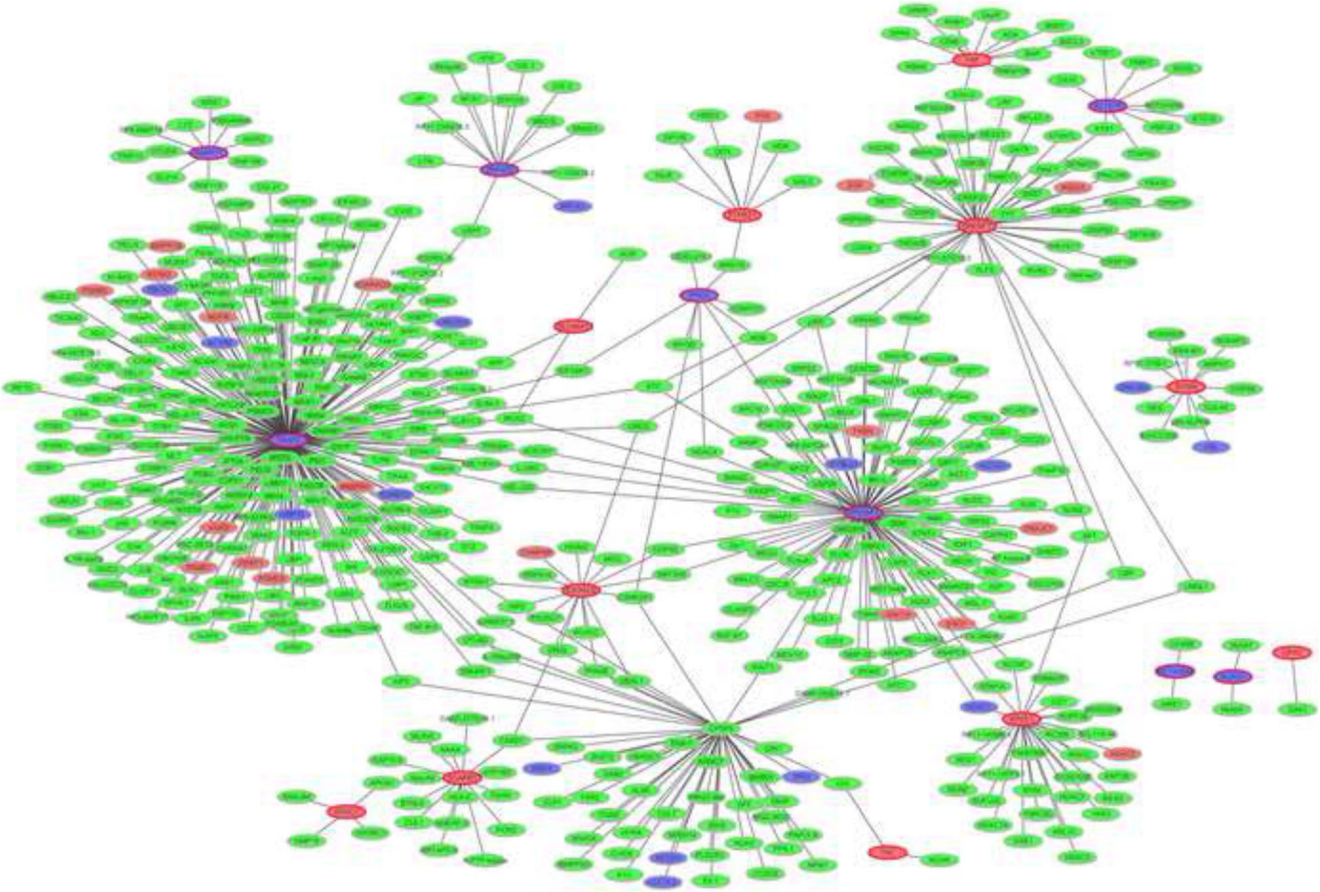
The PPI network of top 30 significantly DEGs in AMD. The *blue ellipses* were represented the proteins encoded by down-regulated DEGs and the *red ellipses* were represented the proteins encoded by up-regulated DEGs. Among which, *ellipses with red border* were derived from the top 30 DEGs in AMD. The *green ellipses* were represented other proteins

The PPI network of top 30 DEGs in AMD was consisted of 559 nodes and 583 edges (Fig. 3). The hub proteins were TRAF6 (degree = 225) and TP53BP1 (degree = 108).

### Transcriptional regulatory network

**Fig. 4:**
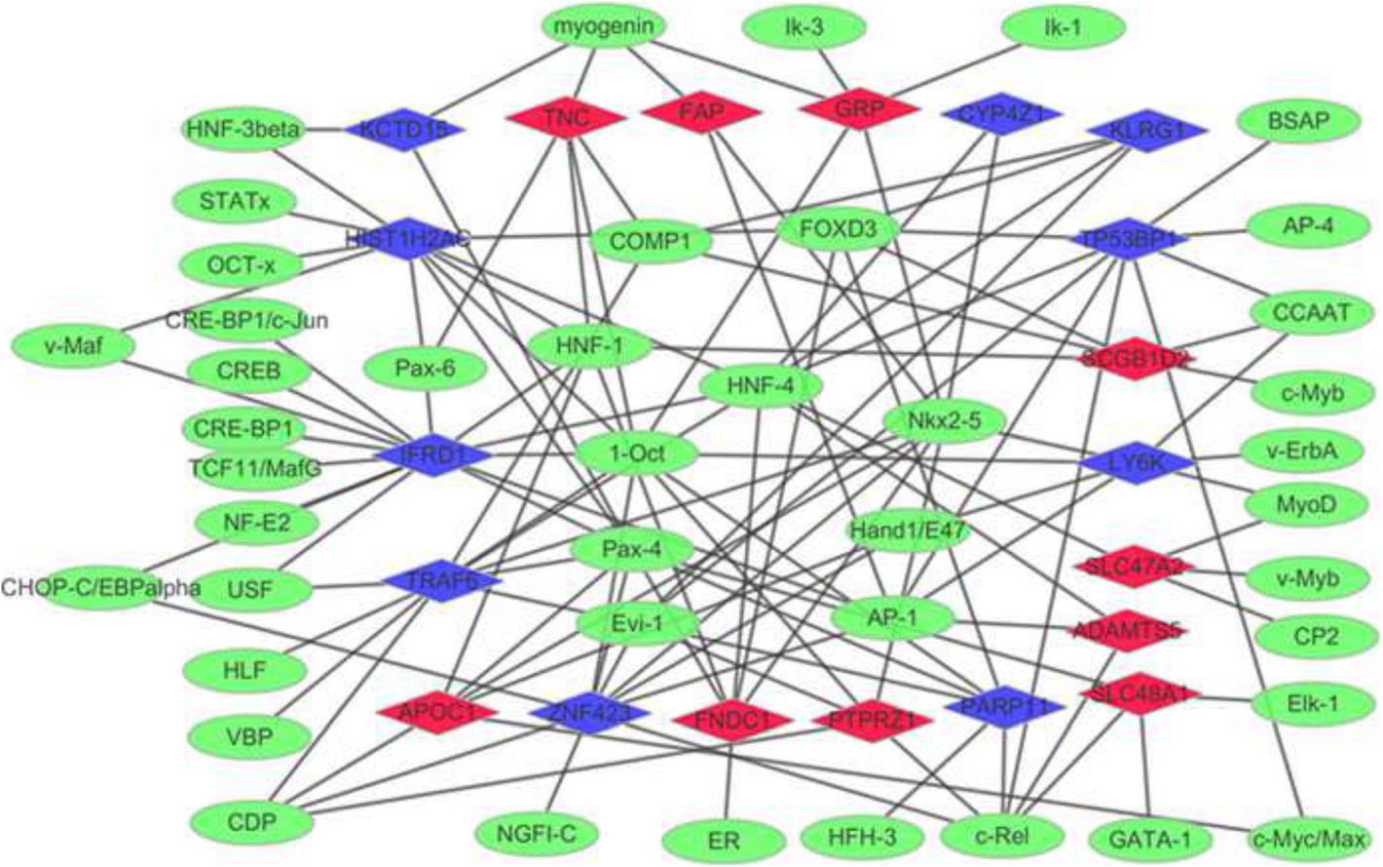
The AMD-specific transcriptional regulatory network. *Red- and blue-color* nodes represent the up- and down-regulated DEGs targeted by TFs, respectively. *Green nodes* denote the TFs which predicted to interact with the corresponding DEGs

Based on TRANSFAC, 43 TFs targeting 20 DEGs (top 10 up-regulated or down-regulated genes) were identified. AMD-specific transcriptional regulatory network was constructed, which consisted of 119 TF-target interactions (Fig. 4). Among of them, c-rel, myogenin, CDP and CCAAT were top 4 TFs covering the most downstream DEGs.

### qRT-PCR confirmation

**Fig. 5:**
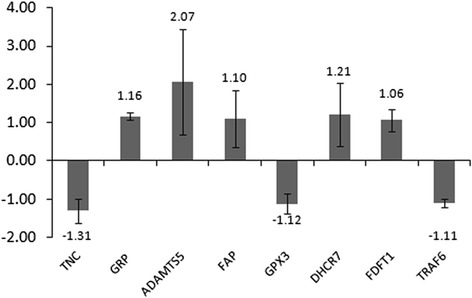
qRT-PCR results of differentially expressed genes in AMD

To verify the expression of integrated analysis, the expression of eight selected genes including Tenascin-C (TNC), fibroblast activation protein alpha (FAP), glutathione peroxidase 3 (GPX3), gastrin-releasing peptide (GRP), ADAM metallopeptidase with thrombospondin type 1 motif 5 (ADAMTS5), TNF receptor associated factor 6 (TRAF6), DHCR7 (7-dehydrocholesterol reductase) and FDFT1 (farnesyl-diphosphate farnesyl transferase 1) were selected to tested by qRT-PCR. Since steroid biosynthesis is a significantly enriched pathway in AMD (FDR = 1.74E-07), the expression of two DEGs (DHCR7 and FDFT1) enriched in steroid biosynthesis were selected to tested by qRT-PCR. The other 6 selected DEGs for qRT-PCR confirmation were derived from the top 30 DEGs. Moreover, TNC was the most significantly DEG in AMD and TRAF6 (degree = 225) was the hub gene based on the AMD-specific PPT network. Based on previous studies, TRAF6 and GPX3 were two AMD-related DEGs [13, 14], two DEGs (FAP and GRP) were choroidal neovascularization-related genes [15, 16] and ADAMTS5 was a retinal pigment epithelium-related DEG [17] which may be involved with AMD. Hence, these six DEGs were selected to be tested by qRT-PCR as well. Except for TNC and GPX3, the expression of other 6 selected genes in qRT-PCR was consistent with our integrated analysis (Fig. 5).

## Discussion

To better reveal the pathogenesis and find more effective treatment for AMD, we performed this integrated analysis between AMD patients and NC groups. 1280 genes across the studies were consistently differentially expressed in AMD (730up-regulated and 550 down-regulated) with P<0.05. Among the top 30 up-regulated or down-regulated DEGs, we selected TNC, FAP, GPX3, GRP, ADAMTS5 and TRAF6 to verify their expression in AMD. Except for TNC and GPX3, the other 4 genes in qRT-PCR played the same pattern with that in our integrated analysis, adding evidences to the reliability of results in the integrated analysis.

As one of extracellular matrix glycol proteins produced by culture fibroblasts, TNC was reported to be up-regulated in AMD [5, 18]. According to our integrated analysis, TNC was increased as well. VEGF is a major pathogenic factor for wet AMD [18] which can promote angiogenesis, vascular permeability and induce macular eduma (ME) [19]. Animal studies have demonstrated that the up-regulation of VEGF is a cause of CNV in the RPE [20]. Since TNC can up-regulate the expression of VEGF, so we concluded that increased TNC may play a role in the process of AMD by regulating CNV. In addition, AMD is associated with degeneration of RPE [21], increased TNC may involve with AMD by repressing the adhesion and migration of RPE. However, the expression of TNC was down-regulated in the blood of AMD patients, larger scale of studies were needed.

FAP is known to be involved in the control of fibroblast growth, which was speculated to be a pioneer for the growth of corneal neovascularization [15]. The up-regulation of FAP detected in this study may be closely associated with AMD by influencing the CNV as well.

To our best knowledge, none of previous studies have reported the association between AMD and GRP. GRP was reported to be involved in the induction of angiogenesis during neuroblastoma progression [16], which showed up-regulation in AMD in our study. Hence, we suspected that increased GRP may play an important role in CNV of AMD.

ADAMTS5 (also known as ADAMTS11) is a member of ADAMTS family. The ADAMTS family has been reported to involve with embryonic development, angiogenesis and cartilage degradation [17]. Bevitt et al. reported the expression of ADAMTS genes in an ocular cell type, ARPE-19 and their regulation by TNF-α. TNF-α is known to play a role in the development of retinal neovascularisation and RPE migration in AMD [17]. Hence, ADAMTS genes may involve with AMD and other retinal pathologies by regulating the proteolytic modification of the retinal extracellular matrix (REM) [17]. According to our study, up-regulated ADAMTS5 was detected in AMD, which supported this hypothesis.

TRAF6 has been reported to involve with the process of AMD by regulating signal transduction pathway [13]. In our study, the expression of TRAF6 was down-regulated. The precise role of TRAF6 in AMD was not clear.

The expression of GPX3 has been reported to be up-regulated in the blood of late AMD patients which was presumably due to oxidative stress [14]. In our integrated analysis, the expression of GPX3 was increased. However, down-regulation of GPX3 was detected in the blood of AMD patients and further research was needed.

After function annotation analysis, we found that steroid biosynthesis was a significantly enriched pathway for DEGs. And previous studies have reported that steroids may play a role for the treatment of AMD [22, 23], while mechanism underlying was not clear [24]. As one of steroids, high level of cholesterol was suspected to increase the risk for AMD by promoting the formation of drusen [25–27], since drusen is a major hallmark of AMD which contains lipid such as cholesterol [28–30]. Both DHCR7 and FDFT1 are associated with cholesterol biosynthesis, which were up-regulated in AMD in our study. DHCR7 catalyzes the reduction of DHC7 to cholesterol and deficiency of DHCR7 results in decreased cholesterol and increased DHC7 levels in serum and tissue [31]. FDFT1 (also called squalene synthase) catalyzes the biosynthesis of squalene, a key cholesterol precursor [26]. So we concluded that both increased DHCR7 and FDFT1 may induce AMD by elevating the expression of cholesterol and formation of drusen.

According to the AMD-specific transcriptional regulatory networks, we identified myogenin was a common mediator of FAP, TNC and GRP, so we concluded that myogenin may be involved in AMD by regulating these target genes. As a member of Rel family, c-rel was reported to be an important mediator of various cytokine stimuli such as TNF-α [17] which suggested that c-rel may involve with the pathological process of AMD.

## Conclusions

In our study, eight DEGs (TNC, FAP, GPX3, GRP, ADAMTS5, TRAF6, DHCR7 and FDFT1) were found to involve with the process of AMD by promoting the formation of drusen, regulating the process of CNV, REM and signal transduction pathway or against with oxidative stress. As a common mediator of TNC, FAP and GRP, myogenin may regulate the process of AMD and c-rel may involve with AMD by regulating TNF-α. Our finding could contribute to understanding the mechanism of AMD in molecular levels, providing clues to potential biomarkers and new therapeutic targets for the disease. The limitation of our study was that the number of samples for qRT-PCR confirmation was small. Hence, studies with larger sample size need to be performed to confirm this conclusion.

## Additional file

Additional file 1: Table S1. The top 15 most significantly enriched GO terms in AMD (DOC 120 kb)

## Abbreviations

ADAMTS5: ADAM metallopeptidase with thrombospondin type 1 motif 5
AMD: Age-related macular degeneration
CNV: choroidal neovascularization
DEGs: differentially expressed genes
DHCR7: 7-dehydrocholesterol reductase
FAP: fibroblast activation protein alpha
FDFT: farnesyl-diphosphate farnesyl transferase 1.
GEO: Gene Expression Omnibus database
GO: Gene Ontology
GPX3: glutathione peroxidase 3
GRP: gastrin-releasing peptide
KEGG: Kyoto Encyclopedia of Genes and Genomes
ME: macular eduma
RPE: retinal pigment epithelium
TFs: transcription factors
TNC: Tenascin-C
TRAF6: TNF receptor associated factor 6
VEGF: vascular endothelial growth factor
