# Supplementary material for: Identification of pathogenic genes and upstream regulators in age-related macular degeneration
Source: BMC Ophthalmol. 2017 Jun 26;17:102. doi: 10.1186/s12886-017-0498-z (PMC5485582; doi:10.1186/s12886-017-0498-z)
Supplement: Additional file 1: Table S1. — The top 15 most significantly enriched GO terms in AMD (DOC 120 kb) [file 12886_2017_498_MOESM1_ESM.doc]

Table S1 The top 15 most significantly enriched GO terms in AMD

| **Items** | **Items_Details** | **Count** | **P_value** | **FDR** | **Genes** |
| --- | --- | --- | --- | --- | --- |
| **Biological process** | |  |  |  |  |
| GO:0006355 | regulation of transcription, DNA-dependent (BP) | 114 | 1.87E-11 | 4.74E-08 | CCAR1,ZNF366,TAF9,ZNF221,C14orf43,JRKL,SND1,PMF1,KLF8,TARDBP,WHSC1L1,CUX2,TIGD6,ZBTB22,FUBP1,BTG1,ZIC1,C11orf30,CNOT1,GABPA,ZNF83,DMTF1,ZNF738,TAF13,TOX2,ACVRL1,ZNF783,GATA4,LHX6,DMRTA1,ZFP161,RUNX1T1,NFYB,MED29,RASL11A,GTF3A,ZNF280D,ZNF568,SCAND1,FLI1,ARID1B,ZNF687,ZNF347,SREBF1,ZNF536,MORF4L1,MAGED1,TSC22D4,GTF2H4,ATXN3,SMARCA4,AHRR,ZNF561,ZNF195,MEF2D,DYNLL1,PDE8B,CCDC59,ZNF582,ZNF239,CHD4,ZNF600,FADS1,ZNF701,CTNND2,MLLT3,HDAC1,NCOA3,ZBTB47,CBL,ZNF44,ZNF669,ZNF177,APBB2,ZBTB44,CCNH,CHD3,MYBBP1A,GRM5,PAX8,ZNF521,ZNF304,ETV5,SALL4,DR1,NLK,ZNF772,ZNF440,CALR,UHRF1,PRKCB,GPBP1L1,WWC1,CCRN4L,ZNF597,SAP30,ZNF276,PSPC1,NR2F2,ZNF517,ELF3,ZNF565,ZNF653,ZRANB2,ZNF813,ZNF426,NFIX,SHOX,ZNF696,OTX2,IFNK,ZNF92,SSX5,SOLH |
| GO:0034641 | cellular nitrogen compound metabolic process (BP) | 28 | 1.48E-09 | 1.25E-06 | HPD,PSMD5,SLC43A2,SLC1A4,BCAT1,ALDH9A1,AFMID,ARG1,PSMB1,SLC7A11,ADC,AGXT,SLC38A2,BCKDHA,PSMD12,GOT2,GPT,QDPR,PSMD1,ACADSB,SLC7A6,AMD1,BCKDHB,PSMB5,PAOX,ALDH4A1,GSTZ1,PSMD3 |
| GO:0006695 | cholesterol biosynthetic process (BP) | 12 | 1.03E-09 | 1.30E-06 | SQLE,INSIG1,HMGCS1,FDFT1,IDI1,EBP,GGPS1,DHCR7,DHCR24,FDPS,MVK,TM7SF2 |
| GO:0006810 | transport (BP) | 53 | 6.51E-09 | 3.30E-06 | APOC1,FADS2,NDUFB9,RBP5,MB,GJB1,SLC4A4,GABRB1,NCBP1,DBI,APOO,SLC37A4,SLC25A4,SORT1,DDX25,ABCB1,LAPTM4B,AP3S1,NXF3,CETP,ABCG2,SLC25A43,CLIC2,AIFM3,HBG1,GJC1,DYNLL1,SLC25A38,ABCA10,CD36,FADS1,ABCA13,GOT2,SEC14L3,SLC25A10,GABRE,SLC48A1,SCARB1,SLC29A4,NCBP2,PCTP,TMEM9,SLC7A6,PTGDS,ACBD5,FADS3,NDUFS1,ACBD3,SEC14L2,TRAPPC4,SLC25A13,PNPT1,SLC10A3 |
| GO:0008285 | negative regulation of cell proliferation (BP) | 37 | 5.49E-09 | 3.48E-06 | PPARG,BTG1,ADORA1,SSTR1,FRZB,FEZF2,ALDH1A2,ACVRL1,PPP2R5C,CDKN2B,EREG,GDF11,CYP27B1,SPRY2,SIRPG,MAP2K1,SFRP2,CDKN3,SOX7,IRF6,KAT2B,FGFRL1,TGFB3,TOB2,TGFB2,ERBB4,SLC9A3R1,AMBRA1,TBRG1,DHCR24,EIF2AK1,DDR1,IL28RA,NOS3,RARG,IFNK,DLG3 |
| GO:0007399 | nervous system development (BP) | 41 | 9.08E-09 | 3.84E-06 | THBS4,GJB1,ADORA1,FEZF2,NUMB,GLDN,FZD9,LHX6,NEDD4,SLIT3,ZEB2,GDF11,ARID1B,PTN,ATXN3,SMARCA4,FGF1,MEF2D,PDPN,ENC1,HMX2,ULK2,CCDC88A,FAM5C,NTRK2,CAMK2B,SPOCK1,CHRNA3,METRN,RGS9,CSPG5,LY6H,ERBB4,CHN1,JAG1,AMBRA1,BRSK2,NR2E1,TRPC5,LIMK1,ZNF423 |
| GO:0045893 | positive regulation of transcription, DNA-dependent (BP) | 44 | 1.68E-08 | 6.10E-06 | HNF1A,PPARG,ZIC3,LEF1,TP53BP1,ZIC1,CD86,LRP5,FEZF2,ACVRL1,GATA4,NFYB,TFAM,PCBD2,SMARCA4,PIAS2,BRCA1,SOX8,SNAI1,SERTAD3,KLF7,HDAC1,IRF6,EGF,NCOA3,SLC25A10,SOX9,TP63,MYSM1,TGFB3,SOST,PAX8,ETV5,ERBB4,MDFIC,CRLF3,NR2F2,BMP7,SEC14L2,ELF3,SERTAD1,LHX2,ZNF423,OTX2 |
| GO:0006629 | lipid metabolic process (BP) | 29 | 2.51E-08 | 7.95E-06 | APOC1,HSPG2,FADS2,GDPD3,PPARG,CUBN,CLU,DEGS1,RBM12,HMGCS1,CETP,SREBF1,CPT1C,PLCZ1,CD36,ACAT2,FADS1,APOE,PLCH1,PNPLA4,SCARB1,LCN12,ACADSB,PTGDS,ALDH3A2,FADS3,BTN2A1,NR2F2,ACOT1 |
| GO:0045892 | negative regulation of transcription, DNA-dependent (BP) | 38 | 1.28E-07 | 3.61E-05 | MECP2,HNF1A,PPARG,LEF1,CUX2,SMURF2,HDAC3,TRAF6,SUDS3,SETD8,EREG,ENO1,ZNF438,ZNF224,TSC22D4,SMARCA4,BRCA1,SFRP2,SOX8,CCDC85B,UIMC1,SOX7,HDAC1,ID1,SNW1,SOX9,TP63,UBP1,GATAD2A,CALR,MDFIC,HEY2,PBXIP1,NR2F2,BMP7,ELF3,CBY1,ZNF423 |
| GO:0007165 | signal transduction (BP) | 79 | 2.39E-07 | 6.06E-05 | ASAP1,ERG,RALGDS,CRHBP,GABRB1,PPARG,UNC5CL,EDARADD,ITPK1,LRRN2,CD244,IFNGR1,RTKN2,GRASP,ADORA1,RXFP1,VAC14,ACVRL1,TRAF6,BAIAP2,TEK,DKK1,PPP2R5C,RASL11A,ROCK1,NGFR,CLIC2,CAMLG,AMELX,RAPGEF3,FGF1,MAP2K1,ITPKA,PDPN,SFRP2,RHPN2,SOX8,TNC,ANGPT4,SLC35B2,CTNND2,PDE7B,ULK2,ARRDC5,CAMK2B,EGF,RGS1,MPP3,PDE2A,DAB2IP,SOX9,PDE1A,TNFRSF10A,PRKCH,HINT1,SPOCK1,RASIP1,CHRNA3,CXCL14,DLL4,CCL8,CNTNAP3,ERBB4,CHN1,GRP,PRKCB,IGFALS,TIE1,CSNK1E,CXCL12,NR2F2,TRH,CXXC5,RSU1,NOS3,TNN,PPP1R12A,LIMK1,RARB |
| GO:0030308 | negative regulation of cell growth (BP) | 17 | 8.55E-07 | 0.000197 | PPARG,BTG1,FRZB,ACVRL1,ENO1,SLIT3,CYP27B1,SMARCA4,NME6,SFRP2,SERTAD3,CCDC85B,DAB2IP,APBB2,TGFB2,CRLF3,TCHP |
| GO:0045944 | positive regulation of transcription from RNA polymerase II promoter (BP) | 46 | 1.04E-06 | 0.000219 | TAF9,HNF1A,TFAP2E,PPARG,ZIC3,LEF1,TP53BP1,GABPA,LRP5,TOX2,TRAF6,GATA4,STAT5B,LMO2,SREBF1,SMARCA4,PIAS2,FGF1,KLF15,MEF2D,BRCA1,SFRP2,SOX8,CSRNP3,HDAC1,NFATC3,KAT2B,NCOA3,DAB2IP,SOX9,TP63,CCNH,TGFB3,IKZF1,PAX8,SALL4,FZD5,UHRF1,CRLF3,GABPB1,BMP7,ELF3,LHX2,NFIX,RARG,OTX2 |
| GO:0006636 | unsaturated fatty acid biosynthetic process (BP) | 4 | 1.83E-06 | 0.000358 | FADS2,DEGS1,FADS1,FADS3 |
| GO:0007155 | cell adhesion (BP) | 44 | 2.05E-06 | 0.000372 | OPCML,THBS4,COL9A1,FAT4,LRRN2,LEF1,DCBLD1,PCDH12,CD209,COL22A1,SCARB2,EDIL3,SDK1,MEGF10,DGCR6,HEPACAM,CLDN10,OMG,AMELX,SIRPG,ITGA5,LRFN3,CD36,PCDH19,F8,TNC,SPP1,PKP3,CTNND2,SCARB1,ITGB4,SPOCK1,CNTNAP3,PPFIBP1,IGFALS,CCR3,CXADR,CXCL12,CADM4,DDR1,MPZL3,CD96,CHST10,COMP |
| GO:0007275 | multicellular organismal development (BP) | 64 | 2.56E-06 | 0.000406 | ERG,HEMGN,TSNAX,EDARADD,SORT1,DDX25,LRP5,FRZB,FEZF2,NUMB,DONSON,GLDN,EDIL3,LHX6,DKK1,LMO2,NGFR,EREG,SLIT3,POMT1,PLCZ1,IFRD1,PAK3,AMELX,SPRY2,FGF1,MEF2D,ENC1,SNAI1,LECT1,CTNND2,HMX2,NTRK2,CAMK2B,ANO1,ID1,HTATIP2,CHST2,ITGB4,IKZF1,OBSCN,METRN,CSPG5,PAX8,ZNF521,TNP2,UPK2,DLL4,FZD5,RPS4Y1,JAG1,AMBRA1,NR2E1,PBXIP1,ELF3,CYLC2,PHC1,ALX3,RTTN,ZNF423,DYNC2LI1,SHOX,MYCNOS,OTX2 |
| **Cellular component** | |  |  |  |  |
| GO:0016020 | membrane (CC) | 302 | 3.46E-34 | 1.53E-31 | ASAP1,TMEM62,TRIP11,FADS2,NDUFB9,VPS18,CHMP4B,ATXN2L,TMEM160,SPAG4,GOLT1A,MANBAL,TMEM80,FAM174A,C1QBP,AKAP7,PAQR6,GABRB1,GDPD3,UNC5CL,LHFPL1,LRRN2,APOO,STOML1,SLC37A4,HSD17B2,SLC47A2,IL22RA1,IFNGR1,EDEM3,NETO2,SLC25A4,SORT1,MXRA7,SQLE,SNN,PTPRZ1,COG1,LRRN1,DCBLD1,INSIG1,SLC27A2,CD209,SLC1A4,ABCB1,IMPAD1,CDC42EP2,SLC18A1,SLC35E3,LAPTM4B,LRP5,CRHR1,TOMM34,FRZB,RXFP1,RPRM,VAC14,TMEM184B,CUBN,HAVCR1,ACVRL1,SLITRK2,TMEM38A,CDC42EP4,AP3S1,HHIPL1,CLU,TOM1L1,ATP2C2,SLC38A6,ZDHHC24,SCARB2,TEK,MUC3A,DEGS1,SDK1,TMED9,TMEM5,RASL11A,TMEM218,TMEM151A,DCT,ROCK1,BSPRY,TMEM206,TMEM177,CRELD1,NPDC1,PKHD1L1,SERAC1,FAM173A,C12orf69,RDH11,SLC25A43,FDFT1,UGT3A2,CYP2B6,SYNPR,HEPACAM,POMT1,CLDN10,FAM57A,CLIC2,SREBF1,CYP27B1,LPGAT1,CPT1C,FKBP7,CKLF,ZFYVE9,CAMLG,SUSD1,VPS39,SLC39A8,GIMAP1,SOAT2,SPRY2,SNX4,CYP4Z1,SIRPG,ARL6IP1,PEX10,FMO5,DPM2,TMEM135,IL20RA,ZDHHC4,MAMDC4,DOLK,SLC38A2,SLC25A38,TBC1D20,ABCA10,LRIG1,FNDC5,HERPUD1,BTNL9,SFT2D3,ST3GAL5,LOXL3,CD36,TMTC4,SLC2A5,C17orf87,SCD,SCGN,PPAPDC1A,SLC35B2,LECT1,FADS1,SCARA3,ATP7A,ATL2,ABCA13,PIGX,PTPLB,LRRC15,MPDU1,RRH,RHBDD1,AGPAT5,ADCK4,ATP5F1,GPM6A,CLEC4D,CCDC88A,CALCRL,TMEM169,C9orf5,PLCH1,TMEM209,AFG3L2,ASPHD1,GRK6,B4GALNT4,NTRK2,PPAPDC2,FAP,RETSAT,TLCD1,EGF,GALNT1,CTLA4,SLC25A10,TMEM132A,DNAJB12,GPR137B,MPP6,COPG2,ST6GALNAC4,CHST2,CYP2W1,SORCS2,SLC48A1,CHST6,SCARB1,GABRP,TNFRSF10A,MRC2,APBB2,CHRNA10,SLC29A4,FXYD3,MFSD3,KLRF1,C6orf64,SPCS1,STX3,TMEM39A,TMEM129,MFF,HS3ST4,B3GALT4,SLC13A5,OAS2,TRPC4,PARL,ITM2A,ELOVL3,LAMP1,TRDN,ITPRIPL1,EBP,TMEM198,TMEM53,JAG1,SLC23A2,SLC9A3R1,SERINC3,GRAMD1C,ATP6AP1,HEG1,CLDND1,NPR3,TMEM9,TAS2R10,C19orf63,CANX,TM6SF1,SLC7A6,C10orf35,ATP5S,WSCD1,DHCR7,PTPRB,PTGDS,VPS33B,ACBD5,P4HTM,TRPC5,FAM162A,ALDH3A2,DHCR24,OPA1,FADS3,CACNG6,NDUFS1,BTN2A1,TSPAN12,ATP10B,ACBD3,CADM4,DNAJC18,MYADM,SLC15A4,ANO3,MARVELD3,TMEM100,DDR1,KCNK16,RNF19A,NAGPA,MPZL3,TSPAN33,COX11,IL28RA,DISP1,SSR1,TXNDC15,COQ10A,TXNDC11,LRRC3B,NPVF,PRRG3,TMEM14B,SLC25A13,TMEM191A,SLC10A3,TM7SF2,MBOAT1,CHST10,BSG,KCTD15,PCSK6,C14orf1,SLC9A8,FOXRED1,TTC19,THSD1,BMPR1B,SLC9A11,RHOBTB2 |
| GO:0005737 | cytoplasm (CC) | 361 | 9.80E-34 | 2.17E-31 | ASAP1,CCAR1,TRIP11,MED27,ERG,HNF1A,HRASLS,RBP5,GART,CHMP4B,TSNAX,HRSP12,SPAG4,UBE4B,DNAJC7,SPAG1,C1QBP,AKAP7,KIAA1199,FGFR1OP,SND1,FGD6,GRK5,EIF3K,PPARG,ERRFI1,NCBP1,PACSIN3,UNC5CL,EDARADD,ZIC3,OLA1,LEF1,SULT1C2,CTSL2,PPA2,PEX7,TPT1,TPD52L2,SMURF2,KIF23,TP53BP1,BTG1,TRIM45,TPP2,DSTN,ZIC1,SULT1E1,GFAP,NANOS1,APC2,GRASP,HOMER1,DDX25,RBM3,APEH,LYPLAL1,PPP4R4,CD209,CNOT1,C11orf21,CDC42EP2,SLC18A1,DMTF1,PGD,CLEC3B,BCAT1,MAGEE1,TOMM34,HSPBAP1,ARHGEF16,FRZB,DDAH1,RPRM,SP7,HDAC3,SDPR,C13orf15,ALDH9A1,COL22A1,CUBN,TUBB2B,SRP72,ALDH1A2,TRAF6,PSMG1,BAIAP2,UBASH3A,METAP2,CDC42EP4,SMU1,TIAM2,MGRN1,DSP,CLU,TOM1L1,UBXN6,STAT5B,FZD9,NXF3,HMGCS1,DCT,CDKN2B,ROCK1,NEDD4,NGFR,BSPRY,ENO1,AFMID,ZEB2,ARG1,PSMB1,FASN,FH,PLEKHA1,MYRIP,SNX3,PRKCG,PLEKHA8,DCTPP1,HEPACAM,CLIC2,COMMD4,SPTBN2,CYP27B1,SGCB,LPGAT1,SYNM,CSRP2BP,AIFM3,FBXL4,MAGED1,PLCZ1,FCGBP,ZFYVE9,VPS39,MAL2,NT5C,ATXN3,PAK3,AHRR,FNDC1,SPRY2,EIF1,SNX4,ARL6IP1,TIPIN,NMBR,BCL2A1,CCT6B,FGF1,MAP2K1,DYNLL1,HSPB8,BRCA1,RYR1,NBPF15,CTNNBIP1,CA1,KATNAL2,FAM40B,AQP11,EXOSC9,MCOLN1,DAG1,SNTB1,PPL,SFRP2,PRC1,TOP1,NEK2,RHPN2,SOX8,ENC1,SNAI1,CDKN3,CA3,TTC8,DCLK2,RAB8A,MAP7,TEX14,ACAT2,CHD4,PLA2G12A,SCGN,PYGL,SPP1,ATP7A,RGS16,GNPDA2,CTNND2,UBE2E3,APOE,S100A13,TEKT2,CCDC85B,QARS,SHROOM3,GAN,MYLK,PEBP1,CCDC88A,PLCH1,FAM5C,SOX7,NUDT6,S100A12,FNIP2,HDAC1,EXOC5,DDX3Y,NFATC3,CAMK2B,ANO1,AARS2,IRF6,BCAS3,CABP7,RGS1,ID1,NCOA3,NFKBIE,PDE2A,DAB2IP,ADRB1,COPG2,CBL,SHPK,HTATIP2,TP63,ZNF44,CARD17,DDIT4,SCARB1,PRKCH,APBB2,HINT1,SPRR2G,RDX,GPT,SPOCK1,APC,C9orf103,RASIP1,TGFB3,GNPDA1,CHD3,IKZF1,MYBBP1A,TOB2,QDPR,OBSCN,ALDH1L1,C18orf10,ADSSL1,CALN1,GRM5,MFF,RGS9,CEP57,DNAJB4,POLR2C,GSDMB,MYO7A,CENPJ,PYGB,NCBP2,OAS2,NUDT14,MED22,CIRBP,RP2,PCTP,FZD5,NLK,TRDN,MCOLN3,ERBB4,TTLL6,CALR,MDFIC,MT4,LY6K,ALS2CL,WIBG,PRKCB,REPS2,GGPS1,OVCA2,SLC23A2,SLC9A3R1,CRYM,PARS2,CRLF3,WWC1,AMBRA1,CANX,TOPBP1,CSNK1E,FAM84B,C6orf108,CXADR,RAB3IP,SERPINB5,PTGDS,VPS33B,HEY2,MAP3K12,DHCR24,VARS,DIDO1,IPPK,PBXIP1,TCHP,PSPC1,PADI2,SEC14L2,ELF3,FDPS,EIF2AK1,CYLC2,TRH,RNF19A,PSMB5,NEK9,PAOX,CXXC5,GSTP1,NOS3,USP25,MVK,ATG5,HNRNPH1,FABP1,C1orf88,MTMR1,ADAM8,PPP1R12A,LIMK1,RPS3,DGCR8,KIAA0753,DYNC2LI1,PNPT1,SETX,GSTZ1,ACOT1,ATF5,ALS2CR12,NOXA1,GSTT1,RPS15A,TTC19,OTX2,MT1F,RARB,FBXL12,ARL4A,DLG3,TRIT1 |
| GO:0005634 | nucleus (CC) | 363 | 3.96E-32 | 5.83E-30 | ZNF366,TRIP11,MED27,NR2C1,TAF9,MECP2,ERG,HNF1A,HIST1H2BL,POLR3H,HEMGN,TSNAX,HRSP12,TFAP2E,UBE4B,NUDT12,ZNF221,DNAJC7,C14orf43,C1QBP,AKAP7,HIST1H2BM,FGFR1OP,MYCT1,JRKL,SND1,PMF1,GRK5,EIF3K,PPARG,ERRFI1,KLF8,NCBP1,ZIC3,LEF1,HNRNPC,TARDBP,RBM6,INTS8,CENPP,GADD45A,WHSC1L1,CUX2,TIGD6,ZBTB22,FUBP1,SMURF2,KIF23,TP53BP1,BTG1,TRIM45,TPP2,SORT1,ZIC1,SULT1E1,SPOPL,C11orf30,DDX25,RBM3,POLR2J,FANCI,GABPA,ZNF83,ZNF18,DMTF1,STK17A,MAGEE1,ZNF738,TAF13,SP7,FEZF2,HDAC3,RBMS2,C13orf15,TOX2,ZSCAN5A,HIST1H2BD,WBP4,ALDH1A2,TRAF6,DONSON,BAIAP2,UBASH3A,TMEM38A,SMU1,LGALS12,ZNF783,MGRN1,CLU,GATA4,UBXN6,STAT5B,SETD8,HIST3H2A,NXF3,PPWD1,SYAP1,LHX6,DMRTA1,RBM12,ZFP161,H1F0,RUNX1T1,NFYB,DHX32,PPP2R5C,MED29,RASL11A,GTF3A,ZNF280D,CDKN2B,LMO2,NGFR,BSPRY,SF3A1,ZNF568,ENO1,AFMID,ZEB2,KIAA0101,PSMB1,DGCR6,PLEKHA1,SCAND1,PRKCG,SP140L,ZNF438,FLI1,ARID1B,EBNA1BP2,ZNF687,ZNF347,CLIC2,SREBF1,ZNF536,MORF4L1,CSRP2BP,AIFM3,FBXL4,ZNF224,PLCZ1,CEBPG,TSC22D4,XRCC2,GTF2H4,C1orf61,IFRD1,NT5C,ATXN3,PCBD2,SMARCA4,PIAS2,AHRR,HIST1H2BH,TIPIN,BCL6B,ZNF561,FGF1,ZNF195,KLF15,MEF2D,MAP2K1,DYNLL1,HSPB8,BRCA1,FAM107A,CTNNBIP1,EXOSC9,DAG1,CCDC59,CPSF3,NUCKS1,C12orf39,PPL,MUSTN1,PRC1,TOP1,NEK2,SOX8,ENC1,SNAI1,ZNF582,ZNF239,ACAT2,CHD4,ZNF600,FADS1,SERTAD3,ZNF701,CSRNP3,PKP3,USP36,CTNND2,HIST1H1C,UBE2E3,VCX3A,S100A13,CCDC85B,HMX2,MLLT3,HNRNPH3,UIMC1,KLF7,SOX7,NUDT6,S100A12,FNIP2,HDAC1,DDX3Y,NFATC3,IRF6,BCAS3,ID1,SNW1,KAT2B,NCOA3,SLC25A10,PDE2A,ZBTB47,PDCD7,ADRB1,CBL,MORC3,SOX9,HTATIP2,TP63,ZNF44,ST6GALNAC4,FHL5,ZNF669,MYSM1,BCL9,CENPH,ZNF177,APBB2,HINT1,GTF3C6,APC,ZBTB44,CCNH,TGFB3,CHD3,IKZF1,MYBBP1A,TOB2,UBP1,RGS9,ZIC4,PAX8,CEP57,HIST1H2BO,DNAJB4,POLR2C,ZNF521,MPO,GATAD2A,NCBP2,OAS2,TNP2,ZNF304,ETV5,SALL4,MED22,CIRBP,CRCP,ACRC,PARL,DR1,NLK,BUB3,GTF3C2,ZNF772,CA9,USP3,ERBB4,ZNF440,CALR,PRPF18,UHRF1,MDFIC,LY6K,PAPOLG,WIBG,PRKCB,IGFALS,OVCA2,GPBP1L1,SLC9A3R1,CRYM,WWC1,TBRG1,TOPBP1,RAD52,CCRN4L,ZNF597,CSNK1E,C6orf108,SAP30,CXADR,PCP4,RAB3IP,NR2E1,PTGDS,HEY2,DDX11,HIST1H2BJ,DHCR24,ZNF276,ARL6IP4,DIDO1,IPPK,CLUAP1,PBXIP1,RPAP1,PSPC1,TACR3,NR2F2,ZNF517,GABPB1,SEC14L2,ELF3,FDPS,ZNF565,ZNF653,TRH,PHC1,PSMB5,ALX3,NEK9,CBY1,CXXC5,HIST1H2BB,NSFL1C,ZRANB2,ZCCHC8,GSTP1,NOS3,DGCR6L,USP25,HEATR1,HNRNPH1,FABP1,ZNF813,RMI1,LHX2,LIMK1,ZNF423,RPS3,DGCR8,SETX,ZNF426,NFIX,SHOX,ATF5,ZNF696,RARG,OTX2,MT1F,ZNF92,RARB,SSX5,ARL4A,HIST1H2AC |
| GO:0016021 | integral to membrane (CC) | 308 | 3.12E-30 | 3.44E-28 | TMEM62,TMEM160,SPAG4,GOLT1A,MANBAL,TMEM80,FAM174A,GJB1,KLRG1,PAQR6,GABRB1,KCNE1,GDPD3,UNC5CL,LHFPL1,SLC30A1,FAT4,LRRN2,APOO,STOML1,SLC37A4,GRM7,CD244,HSD17B2,SLC47A2,IL22RA1,NETO2,SORT1,TBXA2R,SLC43A2,MXRA7,SQLE,SNN,EPCAM,LRRN1,DCBLD1,INSIG1,CD86,SLC27A2,CD209,PRSS8,SLC1A4,ABCB1,IMPAD1,OR51Q1,SLC18A1,SLC35E3,LAPTM4B,ALPL,LRP5,CRHR1,TOMM34,RXFP1,RPRM,TMEM184B,OR6K2,HAVCR1,EMCN,GRIK3,SLITRK2,TMEM38A,OR10P1,GLDN,ATP2C2,SLC38A6,ZDHHC24,SCARB2,CD180,FZD9,TEK,MUC3A,DEGS1,SDK1,PPP2R5C,TMED9,TMEM5,TMEM218,TMEM151A,DCT,TMEM206,MEGF10,TMEM177,CRELD1,ABCG2,NPDC1,PKHD1L1,SERAC1,FAM173A,C12orf69,RDH11,SLC25A43,SLC7A11,FDFT1,UGT3A2,SYNPR,HEPACAM,POMT1,CLDN10,FAM57A,CLIC2,SREBF1,SGCB,LPGAT1,CPT1C,CKLF,CAMLG,SUSD1,SLC39A8,MAL2,GIMAP1,SOAT2,CYP4Z1,CNIH3,SIRPG,ARL6IP1,FMO5,DPM2,TMEM135,GJC1,IL20RA,CLDN3,ZDHHC4,MAMDC4,DOLK,SLC38A2,AQP11,MCOLN1,DAG1,SLC25A38,TBC1D20,ABCA10,LRIG1,ITGA5,PDPN,FNDC5,HERPUD1,BTNL9,SFT2D3,LRFN3,ST3GAL5,CD36,TMTC4,SLC2A5,PCDH19,C17orf87,SCD,PPAPDC1A,SLC35B2,LECT1,FADS1,SCARA3,ATP7A,ATL2,ABCA13,PIGX,PTPLB,CRTAM,LRRC15,MPDU1,RHBDD1,AGPAT5,ADCK4,GPM6A,CLEC4D,ENTPD3,TMEM169,C9orf5,SEC14L3,TMEM209,AFG3L2,ASPHD1,B4GALNT4,PPAPDC2,ANO1,FAP,TLCD1,EGF,CABP7,GALNT1,SLC25A10,TMEM132A,DNAJB12,ST6GALNAC4,CHST2,GABRE,SORCS2,SLC48A1,LRBA,CHST6,FGFRL1,SCARB1,GABRP,TNFRSF10A,MRC2,CHRNA10,SLC29A4,ITGB4,MFSD3,C6orf64,SPCS1,STX3,CHRNA3,TMEM39A,TMEM129,CALN1,GRM5,MFF,HS3ST4,CSPG5,ELTD1,B3GALT4,HRH3,SLC13A5,KCNK3,DLL4,TRPC4,PARL,FZD5,ADCY7,CNTNAP3,ITM2A,ELOVL3,TRDN,ITPRIPL1,MCOLN3,CA9,ERBB4,EBP,TMEM198,TMEM53,SLC23A2,TIE1,ABCB9,SERINC3,GRAMD1C,ATP6AP1,HEG1,CLDND1,NPR3,TMEM9,TAS2R10,GPR146,CCR7,C19orf63,CANX,TM6SF1,SLC7A6,C10orf35,CCR3,WSCD1,DHCR7,KCNK7,ACBD5,P4HTM,FAM162A,OR2T5,ALDH3A2,DHCR24,OPA1,FADS3,CACNG6,BTN2A1,GCGR,TSPAN12,TMC8,ATP10B,ACBD3,SEC14L2,CADM4,DNAJC18,MYADM,SLC15A4,ANO3,MARVELD3,TMEM100,KCNK16,RNF19A,GPR116,NAGPA,MPZL3,TSPAN33,COX11,IL28RA,DISP1,SSR1,TXNDC15,TXNDC11,LRRC3B,PRRG3,TMEM14B,SLC25A13,SLC16A9,TMEM191A,SLC10A3,TREM2,GPR45,MBOAT1,SLC16A11,CHST10,BSG,C14orf1,SLC9A8,FOXRED1,SLC14A1,RARG,THSD1,NPY1R,SLC9A11 |
| GO:0005886 | plasma membrane (CC) | 242 | 2.56E-21 | 2.26E-19 | HSPG2,OPCML,C1QBP,AKAP7,MMP16,GJB1,KLRG1,SLC4A4,GABRB1,KCNE1,GRK5,ERRFI1,PACSIN3,SLC30A1,FAT4,GRM7,CD244,SLC47A2,MYO1G,IFNGR1,PDIA6,SMURF2,RTKN2,AQP5,SORT1,TBXA2R,SLC43A2,APC2,GRASP,HOMER1,ADORA1,SSTR1,EPCAM,PCDH12,CD86,FANCI,CD209,PRSS8,SLC1A4,OR51Q1,CDC42EP2,ALPL,LRP5,CRHR1,MAGEE1,RXFP1,SDPR,OR6K2,ALDH9A1,CUBN,WBP4,EMCN,SRP72,GRIK3,TRAF6,NUMB,MC3R,BAIAP2,OR10P1,MGRN1,GLDN,DSP,CCR10,CD180,FZD9,TEK,LIFR,DKK1,TAC1,NEDD4,NGFR,EREG,PRND,ENO1,MEGF10,ABCG2,FASN,PLEKHA1,SLC7A11,PRKCG,CLDN10,FAM57A,AIFM3,MAGED1,OMG,SLC39A8,MAL2,FNDC1,FOLR1,SPRY2,CNIH3,SIRPG,NMBR,RAPGEF3,BDKRB2,GJC1,MAP2K1,DYNLL1,CLDN3,BRCA1,RYR1,SLC38A2,AQP11,MCOLN1,DAG1,ITGA5,PDPN,PPL,SFRP2,PRC1,LRFN3,CD36,TTC8,RAB8A,SLC2A5,MAP7,PCDH19,F8,PYGL,CTSG,ATP7A,RGS16,CRTAM,GOT2,APOE,CCDC88A,CALCRL,ENTPD3,FNIP2,NFATC3,CAMK2B,ANO1,FAP,EGF,CABP7,PTPRA,RGS1,CTLA4,PDE2A,DAB2IP,ADRB1,MPP6,CBL,GABRE,LRBA,CIDEB,FGFRL1,SCARB1,GABRP,PRKCH,HINT1,CHRNA10,SLC29A4,RDX,ITGB4,TGFB3,GPC3,DOK7,CHRNA3,CALN1,GRM5,RGS9,RAB33A,CSPG5,ELTD1,SLC16A3,DNAJB4,HRH3,SLC13A5,LY6H,UPK2,KCNK3,RP2,CRCP,DLL4,TRPC4,FZD5,ADCY7,CNTNAP3,TRDN,MCOLN3,CA9,ERBB4,PPFIBP1,LY6K,PRKCB,KCNMB4,JAG1,SLC23A2,SLC9A3R1,TIE1,CRYM,ABCB9,SERINC3,WWC1,GPR146,CCR7,LY6G6C,SLC7A6,CCR3,GPC2,FAM84B,CXADR,CD177,MARCKSL1,KCNK7,MAP3K12,OR2T5,TCHP,TACR3,GCGR,RAB26,TSPAN12,RAB22A,DDR1,RAB8B,TRH,KCNK16,GPR116,KCND3,GSTP1,NOS3,GPC1,MTMR1,ARSA,ADAM8,CD96,P2RY2,PNPT1,SLC16A9,TREM2,HSD17B8,GPR45,SLC16A11,BSG,NOXA1,SLC14A1,BMPR1B,NPY1R,ARL4A,DLG3,RHOBTB2 |
| GO:0005783 | endoplasmic reticulum (CC) | 86 | 3.58E-13 | 2.64E-11 | APOC1,FADS2,AGR2,GJB1,HPD,SLC37A4,EDEM3,PDIA6,AQP5,KDELC1,SORT1,SQLE,SULF2,ADORA1,INSIG1,SLC27A2,LRP5,VAC14,CUBN,PSMG1,CLU,DEGS1,TMED9,RDH11,FDFT1,CYP2B6,PTN,POMT1,SREBF1,LPGAT1,AIFM3,FKBP7,CAMLG,GIMAP1,SOAT2,CYP4Z1,ARL6IP1,FMO5,DPM2,DOLK,AQP11,HERPUD1,MCFD2,PLA2G12A,SCD,FADS1,SCARA3,ATP7A,ATL2,PIGX,PTPLB,MPDU1,TRAPPC5,CCDC88A,CALCRL,RETSAT,TMEM132A,PDE2A,CYP2W1,LRBA,RCN2,SPCS1,CSPG5,POFUT2,OAS2,TRAPPC6A,ELOVL3,EBP,CALR,ABCB9,CANX,GPC2,DHCR7,P4HTM,ALDH3A2,DHCR24,FADS3,ATP10B,SSR1,TXNDC11,TRAPPC4,HSP90B1,TM7SF2,PCSK6,C14orf1,GLT25D2 |
| GO:0005739 | mitochondrion (CC) | 105 | 3.47E-12 | 2.19E-10 | NDUFB9,XPNPEP3,TMEM160,HRSP12,AGR2,C1QBP,SND1,DBI,PPA2,SLC25A4,GBAS,SNN,SLC27A2,SCCPDH,BCAT1,TOMM34,DDAH1,ALDH9A1,DSP,CLU,DEGS1,DHX32,SLIT3,TFAM,KIAA0101,FASN,FH,SLC25A43,CYP27B1,CPT1C,AIFM3,PACRG,NT5C,PCBD2,ADC,NME6,C6orf57,AGXT,MAP2K1,DYNLL1,ARMC1,SLC25A38,PPL,AMACR,ACAT2,BCKDHA,IDI1,MPDU1,GOT2,AGPAT5,ADCK4,ATP5F1,PEBP1,NUDT6,AFG3L2,BRP44L,AARS2,SLC25A10,APOA1BP,ALAS2,CA5B,DDIT4,MMAB,QDPR,ALDH1L1,MFF,MPO,OAS2,PARL,ERBB4,NUDT13,HMGCL,PARS2,ACADSB,ATP5S,AGXT2L1,FAM162A,ALDH3A2,VARS,OPA1,TCHP,NDUFS1,ACBD3,FDPS,RAB8B,BCKDHB,NEK9,COX11,PCCB,COQ10A,MRPL32,P2RY2,SLC25A13,PNPT1,ALDH4A1,GSTZ1,HSD17B8,ALDH2,PSTK,ACAD10,BSG,FOXRED1,QRSL1,TTC19,TRIT1 |
| GO:0005615 | extracellular space (CC) | 71 | 4.06E-12 | 2.24E-10 | HSPG2,C5,C1QBP,THBS4,CRHBP,SCGB1D2,TPT1,SULF2,PRSS8,ALPL,CLEC3B,FRZB,GPX3,CLU,DKK1,CETP,TAC1,EREG,SLIT3,ARG1,CP,PKHD1L1,GDF11,PTN,CKLF,C1QTNF7,FGF1,NENF,DAG1,SFRP2,LOXL3,F8,TNC,ANGPT4,MMP7,SPP1,CTSG,APOE,S100A13,PEBP1,FAP,CHIT1,EGF,SPOCK1,TGFB3,GPC3,SOST,METRN,MPO,CXCL14,CCL16,CCL8,TGFB2,CALR,GRP,IGFALS,IL17D,CHI3L1,CCR3,GPC2,CXADR,SERPINB5,PTGDS,CXCL12,PRSS33,BMP7,GPC1,TNN,PCSK6,IFNK,COMP |
| GO:0005887 | integral to plasma membrane (CC) | 84 | 5.38E-12 | 2.64E-10 | FADS2,OPCML,MMP16,SLC4A4,GABRB1,GRM7,IFNGR1,AQP5,SLC25A4,TBXA2R,HOMER1,ADORA1,SSTR1,GBAS,PTPRZ1,PCDH12,SLC1A4,CRHR1,GRIK3,ACVRL1,NUMB,MC3R,CCR10,SCARB2,TEK,LIFR,DEGS1,TMEM5,NGFR,EREG,SGCB,FOLR1,NMBR,BDKRB2,CLDN3,RYR1,MCOLN1,PDPN,ST3GAL5,CD36,RRH,CALCRL,NTRK2,PTPRA,MPP3,CTLA4,GPR137B,ADRB1,GABRE,CIDEB,SCARB1,FXYD3,KLRF1,GPC3,GRM5,CSPG5,SLC16A3,HRH3,UPK2,KCNK3,LAMP1,EBP,KCNMB4,JAG1,SLC23A2,TIE1,SLC7A6,CCR3,CXADR,PTPRB,TRPC5,TACR3,BTN2A1,TSPAN12,DDR1,GPC1,ADAM8,CD96,P2RY2,SLC25A13,TM7SF2,SLC14A1,BMPR1B,NPY1R |
| GO:0005829 | cytosol (CC) | 141 | 1.54E-11 | 6.80E-10 | GART,CHMP4B,RALGDS,AKAP7,FGFR1OP,PMF1,EIF3K,PPARG,NCBP1,ITPK1,HPD,CENPP,SULT1C2,PSMD5,PEX7,SMURF2,KIF23,TPP2,SULT1E1,MYL3,CNOT1,PGD,BCAT1,ARHGEF16,SDPR,ALDH9A1,CUBN,SRP72,ALDH1A2,TRAF6,BAIAP2,UBASH3A,TIAM2,MGRN1,CLU,TOM1L1,STAT5B,RPL26,HMGCS1,DCT,CDKN2B,ROCK1,NEDD4,NGFR,ENO1,AFMID,ARG1,PKHD1L1,PSMB1,FASN,PRKCG,DCTPP1,SPTBN2,AIFM3,HBG1,NT5C,ADC,SPRY2,ARL6IP1,RPS23,FGF1,MAP2K1,DYNLL1,PDE8B,RYR1,CTNNBIP1,AQP11,EXOSC9,NEK2,ALDOA,DOCK6,IDI1,DOHH,PYGL,PSMD12,ATP7A,S100A13,QARS,PDE7B,MYLK,CCDC88A,MYL6B,PLCH1,S100A12,HDAC1,EXOC5,NFATC3,CAMK2B,IRF6,PDE2A,CBL,PDE1A,TP63,CIDEB,CENPH,RPL39,PRKCH,GPT,QDPR,OBSCN,ADSSL1,CEP57,SUGT1,MYO7A,CENPJ,NCBP2,PHKG1,OAS2,PCTP,BUB3,PSMD1,CALR,CHN1,PRKCB,GGPS1,RPS4Y1,CSNK1E,PCP4,RAB3IP,MAP3K12,DHCR24,AMD1,PBXIP1,FDPS,PSMB5,RSU1,GSTP1,NOS3,MVK,FABP1,LIMK1,RPS3,GSTZ1,HSP90B1,ACOT1,GSTT1,RPS15A,NPY1R,PSMD3,GBE1,RHOBTB2 |
| GO:0005576 | extracellular region (CC) | 129 | 2.26E-11 | 9.08E-10 | APOC1,HSPG2,C5,AGR2,THBS4,MMP16,COL9A1,C3orf39,CRHBP,SCGB1D2,APOO,ISM1,CD209,PRSS8,CLEC3B,FRZB,COL22A1,EMCN,GPX3,HHIPL1,CLU,LIFR,EDIL3,MUC3A,CHADL,DKK1,KLK11,CETP,TAC1,NGFR,EREG,SLIT3,CP,GDF11,LYZL1,PTN,IGHG1,TEX264,OBP2B,FCGBP,CKLF,FNDC1,AMELX,FOLR1,C1QTNF7,C6orf57,FGF1,NENF,DAG1,C12orf39,SFRP2,MCFD2,LOXL3,ALDOA,F8,TNC,ANGPT4,MMP7,PLA2G12A,SCGN,LECT1,SPP1,CTSG,MMP28,APOE,S100A13,PCOLCE2,OLFML2B,FAM55C,PGLYRP1,FAM5C,S100A12,ADAMTS10,CHIT1,EGF,GALNT1,APOA1BP,SPOCK1,C17orf67,TGFB3,GPC3,SOST,METRN,HS3ST4,CXCL14,CCL16,LCN12,CCL8,CNTNAP3,TGFB2,COL9A2,PAPLN,CALR,GRP,LY6K,DHRS13,C5orf46,IGFALS,JAG1,IL17D,C2orf69,HEG1,CHI3L1,C19orf63,KRTDAP,GPC2,CXADR,SAAL1,SERPINB5,PTGDS,CXCL12,PRSS33,BMP7,OTOS,DDR1,TRH,GPC1,TNN,ADAMTS5,NPVF,PRRG3,TREM2,WFDC13,PCSK6,ACRBP,TFPI2,THSD1,IFNK,COMP |
| GO:0005622 | intracellular (CC) | 128 | 2.31E-10 | 8.50E-09 | ZNF366,ZNF608,ZNF221,RALGDS,AKAP7,FGD6,KLF8,ITPK1,ZIC3,OLA1,RBM6,ZBTB22,SMURF2,RTKN2,TP53BP1,TRIM45,DSTN,ZIC1,RASGEF1A,RPS4Y2,CD209,ZNF83,ZNF18,ARHGEF16,ZNF738,SP7,FEZF2,ZSCAN5A,ZNF783,NXF3,RPL26,ZFP161,NFYB,GTF3A,ZNF280D,NEDD4,BSPRY,ZNF568,ZEB2,ZFR2,ZFC3H1,RDH11,ZNF438,ARID1B,ZNF687,ZNF347,CLIC2,ZNF536,GTPBP6,ZNF224,SIRPG,BCL6B,RPS23,ZNF561,RAPGEF3,ZNF195,KLF15,HSPB8,BRCA1,TBC1D20,RHPN2,SNAI1,ZNF582,ZNF239,ZNF600,PLA2G12A,RBCK1,ZNF701,CARD16,MRPS17,KLF7,SEC14L3,SOX7,SLC25A10,ZBTB47,DAB2IP,ZNF44,CARD17,ZNF669,CIDEB,DDIT4,ZNF177,RPL39,APBB2,ZBTB44,IKZF1,OBSCN,CSPG5,ZIC4,ZNF521,ZNF304,SALL4,DR1,ZNF772,ZNF440,CALR,CHN1,ALS2CL,RPS4Y1,CCR7,TOPBP1,CARD18,ZNF597,ZNF276,IPPK,ZNF517,SEC14L2,ZNF565,ZNF653,TBC1D16,ZRANB2,TRIM60,GSTP1,ZNF813,LHX2,ZNF423,RPS3,DGCR8,ZNF426,NFIX,ZNF696,RPS15A,ZNF92,NUDT5,SSX5,ARL4A,RHOBTB2,SOLH |
| GO:0005794 | Golgi apparatus (CC) | 73 | 4.75E-09 | 1.61E-07 | TRIP11,GOLT1A,FGD6,HPD,SORT1,APC2,SULF2,COG1,IMPAD1,SDPR,CUBN,AP3S1,TOM1L1,H1F0,TMED9,ROCK1,FASN,SREBF1,TIPIN,MAP2K1,CA1,MCFD2,ST3GAL5,CD36,RAB8A,PLA2G12A,SLC35B2,SCARA3,ATP7A,APOE,TRAPPC5,PEBP1,CCDC88A,B4GALNT4,CABP7,GALNT1,CTLA4,NCOA3,NFKBIE,TMEM132A,PDE2A,COPG2,TP63,ST6GALNAC4,CHST2,LRBA,CHST6,RDX,RASIP1,CALN1,HS3ST4,CSPG5,CEP57,CXCL14,B3GALT4,FZD5,TRAPPC6A,CALR,MDFIC,SERINC3,WWC1,PTGDS,DHCR24,ACBD3,ELF3,NAGPA,CBY1,NSFL1C,NOS3,C1orf88,TRAPPC4,CHST10,SLC9A8 |
| GO:0005789 | endoplasmic reticulum membrane (CC) | 52 | 1.21E-07 | 3.81E-06 | FADS2,GJB1,HPD,SLC37A4,HSD17B2,EDEM3,SORT1,SQLE,INSIG1,SLC27A2,DEGS1,TMED9,RDH11,FDFT1,CYP2B6,POMT1,SREBF1,LPGAT1,CPT1C,GIMAP1,SOAT2,CYP4Z1,DPM2,GJC1,DOLK,HERPUD1,SCD,FADS1,SCARA3,ATL2,PIGX,PTPLB,MPDU1,RETSAT,TMEM132A,CYP2W1,FXYD3,SPCS1,CSPG5,ELOVL3,EBP,CANX,DHCR7,P4HTM,ALDH3A2,DHCR24,FADS3,SSR1,TXNDC11,HSP90B1,TM7SF2,C14orf1 |
| GO:0048471 | perinuclear region of cytoplasm (CC) | 38 | 3.45E-07 | 1.02E-05 | CCAR1,TSNAX,FGFR1OP,TPD52L2,SORT1,NANOS1,APC2,GRASP,MAGEE1,TRAF6,CLU,FZD9,NEDD4,BSPRY,PLCZ1,MAL2,MAP2K1,RHPN2,CDKN3,RAB8A,MAP7,SPP1,ATP7A,S100A13,CABP7,GALNT1,CTLA4,NFKBIE,PDE2A,RASIP1,CALN1,CALR,WWC1,PTGDS,VPS33B,RAB8B,HSP90B1,MT1F |
| **Molecular function** | |  |  |  |  |
| GO:0005515 | protein binding (MF) | 319 | 3.84E-33 | 3.70E-30 | ASAP1,CCAR1,TRIP11,HSPG2,NR2C1,TAF9,MECP2,ERG,HNF1A,VPS18,C20orf94,HEMGN,CHMP4B,AGR2,C1QBP,FGFR1OP,PMF1,SLC4A4,GRK5,PPARG,ERRFI1,NCBP1,PACSIN3,ZIC3,LEF1,HNRNPC,TARDBP,INTS8,CD244,GADD45A,PSMD5,TPD52L2,SMURF2,KIF23,AQP5,TP53BP1,BTG1,SLC25A4,TBXA2R,NANOS1,APC2,ADORA1,GBAS,EPCAM,COG1,INSIG1,POLR2J,CD86,FANCI,PPP4R4,CNOT1,PRSS8,GABPA,ABCB1,CDC42EP2,LAPTM4B,LRP5,CRHR1,ARHGEF16,TAF13,HDAC3,SDPR,ACVRL1,TRAF6,PSMG1,MC3R,BAIAP2,AP3S1,MGRN1,DSP,CLU,TOM1L1,ATP2C2,SUDS3,GATA4,UBXN6,STAT5B,SETD8,TEK,NXF3,DKK1,RUNX1T1,NFYB,PPP2R5C,TMED9,MED29,CDKN2B,ROCK1,LMO2,NEDD4,NGFR,SF3A1,EREG,ENO1,ABCG2,TFAM,PSMB1,ZFC3H1,FASN,PLEKHA1,MYRIP,SLC7A11,SNX3,ARID1B,IGHG1,SREBF1,COMMD4,MORF4L1,SYNM,CSRP2BP,ZNF224,MAGED1,CEBPG,FCGBP,ZFYVE9,TSC22D4,CAMLG,GTF2H4,MAL2,ATXN3,PCBD2,SMARCA4,PIAS2,AMELX,SPRY2,SNX4,SIRPG,ARL6IP1,TIPIN,RPS23,PEX10,BCL2A1,FGF1,DPM2,BDKRB2,AGXT,MEF2D,MAP2K1,DYNLL1,HSPB8,BRCA1,CTNNBIP1,DAG1,SNTB1,CCDC59,CPSF3,ITGA5,PPL,PRC1,TOP1,NEK2,SNAI1,CDKN3,TTC8,RAB8A,F8,CHD4,FAM124B,C17orf87,BCKDHA,FBXL8,DOHH,RBCK1,SERTAD3,ATP7A,RGS16,ATL2,PTPLB,CTNND2,APOE,S100A13,CCDC85B,MLLT3,GAN,ATP5F1,MYLK,ULK2,RNF126,UIMC1,MYL6B,CALCRL,S100A12,FNIP2,HDAC1,FAP,IRF6,EGF,ID1,SNW1,KAT2B,CTLA4,NCOA3,SLC25A10,PDE2A,APOA1BP,PPP2R3A,ADRB1,MPP6,CBL,SOX9,ALAS2,HTATIP2,TP63,BCL9,CENPH,TNFRSF10A,APBB2,RDX,GTF3C6,ITGB4,DDX18,CCNH,TGFB3,GPC3,CHD3,STX3,OBSCN,SOST,RAB33A,CSPG5,PAX8,CEP57,DNAJB4,MYO7A,CENPJ,GATAD2A,NCBP2,NUDT14,MED22,CIRBP,RP2,TRPC4,FZD5,DR1,NLK,ITM2A,BUB3,ELOVL3,TGFB2,ERBB4,CALR,UHRF1,MDFIC,WIBG,PRKCB,REPS2,KCNMB4,SLC9A3R1,TIE1,WWC1,TBRG1,CANX,TOPBP1,RAD52,CSNK1E,FAM84B,SAP30,CXADR,PTPRB,RAB3IP,SERPINB5,VPS33B,HEY2,MAP3K12,DDX11,TRPC5,OPA1,CLUAP1,PBXIP1,TCHP,PSPC1,NDUFS1,NR2F2,ATP10B,GABPB1,BMP7,ELF3,RAB22A,DDR1,BCKDHB,MPZL3,PSMB5,SERTAD1,NEK9,CBY1,ZRANB2,IL28RA,ZCCHC8,GSTP1,NOS3,USP25,ATG5,HNRNPH1,PPM1B,C1orf88,RMI1,ADAM8,TRAPPC4,PPP1R12A,LIMK1,ZNF423,RPS3,SHFM1,DGCR8,PNPT1,GSTZ1,HSP90B1,HSD17B8,SHOX,ATF5,NOXA1,RARG,TTC19,BMPR1B,OTX2,FBXL12,ARL4A,DLG3,COMP |
| GO:0003677 | DNA binding (MF) | 146 | 9.55E-20 | 4.60E-17 | ZNF366,NR2C1,TAF9,MECP2,ERG,HNF1A,HIST1H2BL,POLR3H,TSNAX,TFAP2E,ZNF221,C14orf43,HIST1H2BM,JRKL,PPARG,KLF8,LEF1,TARDBP,RBM6,CUX2,TIGD6,ZBTB22,FUBP1,ZIC1,POLR2J,GABPA,ZNF83,ZNF18,DMTF1,ZNF738,TAF13,SP7,HDAC3,FEZF2,TOX2,ZSCAN5A,HIST1H2BD,ZNF783,GATA4,HIST3H2A,ZFP161,H1F0,RUNX1T1,NFYB,GTF3A,ZNF280D,LMO2,ZNF568,ENO1,TFAM,SP140L,ZNF438,FLI1,ARID1B,ZNF687,ZNF347,SREBF1,ZNF536,ZNF224,CEBPG,XRCC2,SMARCA4,PIAS2,AHRR,HIST1H2BH,BCL6B,ZNF561,ZNF195,KLF15,MEF2D,BRCA1,TOP1,SOX8,ZNF582,ZNF239,CHD4,ZNF600,RBCK1,ZNF701,CSRNP3,HIST1H1C,KLF7,SOX7,DDX3Y,NFATC3,IRF6,ZBTB47,SOX9,TP63,ZNF44,ZNF669,MYSM1,ZNF177,GTF3C6,ZBTB44,CHD3,IKZF1,MYBBP1A,UBP1,MCM9,ZIC4,PAX8,HIST1H2BO,POLR2C,ZNF521,TNP2,ZNF304,ETV5,SALL4,DR1,GTF3C2,ZNF772,PPFIBP1,ZNF440,CALR,UHRF1,GPBP1L1,TBRG1,TOPBP1,RAD52,ZNF597,SAP30,NR2E1,HEY2,DDX11,HIST1H2BJ,ZNF276,PBXIP1,RPAP1,NR2F2,ZNF517,ZNF565,ZNF653,PHC1,CXXC5,HIST1H2BB,ZNF813,SETX,ZNF426,NFIX,ZNF696,RARG,OTX2,ZNF92,RARB,HIST1H2AC |
| GO:0046872 | metal ion binding (MF) | 192 | 3.68E-16 | 1.18E-13 | ASAP1,ZNF366,NR2C1,ZNF608,VPS18,GART,XPNPEP3,NUDT12,ZNF221,MB,COL9A1,RNF214,FGD6,GDPD3,PPARG,KLF8,ZIC3,HPD,WHSC1L1,ZBTB22,TRIM45,ZIC1,NANOS1,ZFYVE21,CD209,ZNF83,IMPAD1,ZNF18,ALPL,AGAP10,PHF14,RXFP1,DDAH1,SP7,FEZF2,ZSCAN5A,WBP4,ACVRL1,RNF213,TRAF6,METAP2,MGRN1,ATP2C2,ZDHHC24,GATA4,LHX6,DMRTA1,ZFP161,RUNX1T1,GTF3A,ZNF280D,DCT,ROCK1,LMO2,LUC7L2,BSPRY,ZNF568,ZEB2,ARG1,CP,ZFC3H1,MYRIP,PRKCG,SP140L,ZNF438,CYP2B6,POMT1,ZNF687,ZNF347,CYP27B1,ZNF536,AIFM3,ZNF224,ZFYVE9,HBG1,NT5C,PAK3,NME6,PIAS2,CYP4Z1,ZCCHC3,BCL6B,PEX10,ZNF561,ZNF195,KLF15,ZDHHC4,PDE8B,BRCA1,ARMC1,CA1,NENF,CPSF3,NEK2,SNAI1,CA3,LOXL3,ZNF582,ZNF239,F8,CHD4,MMP7,BCKDHA,ZNF600,IDI1,DOHH,RBCK1,ZNF701,ATP7A,PDE7B,MYLK,RNF126,KLF7,AFG3L2,ADAMTS10,AARS2,PDE2A,ZBTB47,MORC3,PDE1A,TP63,CA5B,ZNF44,FHL5,CYP2W1,ZNF669,MYSM1,ZNF177,PRKCH,ZBTB44,CHD3,IKZF1,OBSCN,ZIC4,ZNF521,MPO,GATAD2A,TNP2,ZNF304,SALL4,NUDT14,ADCY7,ZNF772,CA9,USP3,NUDT13,ZNF440,CHN1,UHRF1,MT4,PAPOLG,PRKCB,GGPS1,AGAP7,HMGCL,ZNF597,SAP30,STK32C,NR2E1,ZNF276,DIDO1,NDUFS1,NR2F2,ZNF517,FDPS,ZNF565,ZNF653,RNF19A,KCND3,PHC1,NEK9,CXXC5,ZRANB2,ZCCHC8,TRIM60,NOS3,ZNF813,ADAMTS5,LHX2,LIMK1,ZNF423,DGCR8,ZNF426,ZNF696,RARG,BMPR1B,MT1F,ZNF92,RARB,AGAP8,TRIT1,SOLH |
| GO:0008270 | zinc ion binding (MF) | 130 | 2.85E-11 | 6.88E-09 | ASAP1,ZNF366,NR2C1,ZNF608,ZNF221,MMP16,RNF214,PPARG,KLF8,ZIC3,RBM6,WHSC1L1,ZBTB22,TRIM45,ZIC1,NANOS1,ZNF83,ZNF18,AGAP10,PHF14,SP7,FEZF2,ZSCAN5A,WBP4,RNF213,TRAF6,MGRN1,ZDHHC24,GATA4,LHX6,ZFP161,RUNX1T1,GTF3A,ZNF280D,LMO2,BSPRY,ZNF568,ZEB2,ZFR2,FASN,MYRIP,PRKCG,ZNF438,ZNF687,ZNF347,ZNF536,ZNF224,PIAS2,ZCCHC3,BCL6B,PEX10,ZNF561,ZNF195,KLF15,ZDHHC4,BRCA1,CA1,SNAI1,CA3,ZNF582,ZNF239,CHD4,MMP7,ZNF600,RBCK1,ZNF701,MMP28,S100A13,PGLYRP1,RNF126,KLF7,AFG3L2,S100A12,ADAMTS10,ZBTB47,CBL,MORC3,CA5B,ZNF44,FHL5,ZNF669,ZNF177,ZBTB44,CHD3,IKZF1,ZIC4,ZNF521,GATAD2A,OAS2,TNP2,ZNF304,SALL4,ZNF772,CA9,USP3,PAPLN,ZNF440,CALR,UHRF1,MT4,PRKCB,AGAP7,ZNF597,NR2E1,ZNF276,DIDO1,NR2F2,ZNF517,ZNF565,ZNF653,RNF19A,PHC1,CXXC5,ZRANB2,ZCCHC8,TRIM60,ZNF813,ADAMTS5,ADAM8,LHX2,LIMK1,ZNF423,ZNF426,ZNF696,RARG,MT1F,ZNF92,RARB,AGAP8,SOLH |
| GO:0000166 | nucleotide binding (MF) | 136 | 1.83E-10 | 3.53E-08 | TAF9,GART,SPAG1,GRK5,ITPK1,OLA1,HNRNPC,TARDBP,RBM6,HSD17B2,MYO1G,KIF23,DDX25,RBM3,SLC27A2,SCCPDH,ABCB1,STK17A,AGAP10,RBMS2,TUBB2B,ACVRL1,RNF213,ATP2C2,TEK,NXF3,RBM12,DHX32,RASL11A,ROCK1,ABCG2,FASN,RDH11,PRKCG,DCTPP1,GTPBP6,SMARCA4,PAK3,NME6,CCT6B,RAPGEF3,GIMAP6,RBM46,RBM7,MAP2K1,ITPKA,DDX60L,KATNAL2,ABCA10,TOP1,NEK2,DCLK2,RAB8A,TEX14,CHD4,PYGL,ATP7A,ATL2,ABCA13,UBE2E3,UBE2D3,QARS,HNRNPH3,MYLK,ULK2,PEBP1,ENTPD3,AFG3L2,DDX3Y,GRK6,NTRK2,MAPK4,CAMK2B,AARS2,RPS6KL1,PDE2A,SHPK,MMAB,PRKCH,DDX18,C9orf103,CHD3,QDPR,OBSCN,ALDH1L1,MCM9,ADSSL1,RAB33A,MYO7A,NCBP2,PHKG1,OAS2,CIRBP,RP2,CRCP,ADCY7,NLK,ERBB4,PAPOLG,PRKCB,DHRS13,TIE1,ABCB9,PARS2,ATP6AP1,BRSK2,CSNK1E,STK32C,KIAA0430,MAP3K12,DDX11,RBM33,VARS,OPA1,IPPK,PSPC1,RAB26,ATP10B,EIF2AK1,RAB22A,DDR1,RAB8B,NEK9,MVK,PCCB,HNRNPH1,LIMK1,SETX,HSP90B1,HSD17B8,PSTK,QRSL1,BMPR1B,ARL4A,TRIT1,RHOBTB2 |
| GO:0005488 | binding (MF) | 63 | 5.02E-10 | 8.07E-08 | ARMCX5,DNAJC7,SPAG1,KLRG1,SCGB1D2,EIF3K,NCBP1,DBI,PSMD5,APC2,PPP4R4,CD209,CLEC3B,TOMM34,VAC14,SRP72,PPP2R5C,NGFR,SERAC1,SLC25A43,SP140L,ARID1B,TTC7A,KIAA0664,C17orf66,IFRD1,ARMC1,HEATR7A,TTC30B,TTC8,TMTC4,TNC,PKP3,CTNND2,TTC28,TRAPPC5,CLEC4D,TTC15,SLC25A10,COPG2,LRBA,MRC2,RDX,APC,USP35,KLRF1,MYBBP1A,SUGT1,MYO7A,LCN12,CNTNAP3,PSMD1,TRAPPC6A,ARMC9,SAAL1,PTGDS,ACBD5,ACBD3,RTDR1,RTTN,HEATR1,SLC25A13,PSMD3 |
| GO:0005509 | calcium ion binding (MF) | 57 | 3.49E-09 | 4.80E-07 | CCAR1,THBS4,MMP16,FAT4,GRM7,TPT1,EDEM3,MYL3,SULF2,PCDH12,CLEC3B,CUBN,CIB4,STAT5B,EDIL3,SLIT3,CRELD1,EFHC1,PLCZ1,FKBP7,SUSD1,EGFL8,RYR1,DAG1,MCFD2,PCDH19,PLA2G12A,SCGN,MMP28,S100A13,S100A2,MYL6B,PLCH1,S100A12,EGF,CABP7,C1orf87,PPP2R3A,CBL,RCN2,SPOCK1,CALN1,ELTD1,DLL4,CALR,REPS2,JAG1,HEG1,CANX,P4HTM,PADI2,ARSA,ADAM8,PRRG3,SLC25A13,HSP90B1,COMP |
| GO:0003700 | sequence-specific DNA binding transcription factor activity (MF) | 67 | 4.59E-08 | 5.53E-06 | NR2C1,ERG,HNF1A,TFAP2E,PPARG,ZIC3,LEF1,TARDBP,CUX2,FUBP1,ZIC1,GABPA,ZNF83,ZNF18,DMTF1,TAF13,ZSCAN5A,GATA4,STAT5B,LHX6,RUNX1T1,NFYB,ENO1,ZEB2,TFAM,SCAND1,FLI1,SREBF1,TSC22D4,GTF2H4,KLF15,MEF2D,SOX8,RBCK1,CSRNP3,HMX2,KLF7,SOX7,HDAC1,NFATC3,IRF6,CBL,SOX9,TP63,IKZF1,UBP1,PAX8,GATAD2A,ETV5,UHRF1,CCRN4L,NR2E1,HEY2,NR2F2,GABPB1,ELF3,ALX3,ZRANB2,LHX2,NFIX,SHOX,ATF5,RARG,OTX2,ZNF92,RARB,SOLH |
| GO:0016787 | hydrolase activity (MF) | 70 | 8.67E-08 | 8.36E-06 | HRSP12,NUDT12,SPAG1,GDPD3,ITPK1,OLA1,PPA2,SULF2,DDX25,PTPRZ1,APEH,LYPLAL1,ABCB1,IMPAD1,ALPL,DDAH1,HDAC3,ATP2C2,DHX32,AFMID,ARG1,DCTPP1,PLCZ1,NT5C,PDE8B,DDX60L,KATNAL2,CPSF3,CDKN3,CHD4,PLA2G12A,IDI1,PPAPDC1A,ATP7A,ATL2,GNPDA2,PDE7B,ENTPD3,PLCH1,NUDT6,HDAC1,DDX3Y,PPAPDC2,CTDSPL2,PTPRA,PDE2A,PDE1A,PNPLA4,HINT1,DDX18,GNPDA1,CHD3,PM20D2,NUDT14,USP3,NUDT13,OVCA2,PTPRB,ATP10B,PADI2,NAGPA,MPPED1,PPM1B,MTMR1,ARSA,PNPT1,SETX,ACAD10,ACOT1,NUDT5 |
| GO:0005524 | ATP binding (MF) | 96 | 8.23E-08 | 8.82E-06 | TAF9,GART,GRK5,ITPK1,OLA1,MYO1G,KIF23,DDX25,SLC27A2,ABCB1,STK17A,ACVRL1,RNF213,ATP2C2,TEK,DHX32,ROCK1,ABCG2,PRKCG,XRCC2,SMARCA4,PAK3,NME6,CCT6B,MAP2K1,ITPKA,DDX60L,KATNAL2,ABCA10,TOP1,NEK2,DCLK2,TEX14,CHD4,PYGL,ATP7A,ABCA13,UBE2E3,UBE2D3,QARS,MYLK,ULK2,PEBP1,ENTPD3,AFG3L2,DDX3Y,GRK6,NTRK2,MAPK4,CAMK2B,AARS2,RPS6KL1,MORC3,SHPK,MMAB,PRKCH,DDX18,C9orf103,CHD3,OBSCN,MCM9,MYO7A,PHKG1,OAS2,RP2,ADCY7,NLK,ERBB4,TTLL6,PAPOLG,PRKCB,TIE1,ABCB9,PARS2,ATP6AP1,BRSK2,CSNK1E,STK32C,MAP3K12,DDX11,VARS,IPPK,ATP10B,EIF2AK1,DDR1,NEK9,MVK,PCCB,LIMK1,P2RY2,SETX,HSP90B1,PSTK,QRSL1,BMPR1B,TRIT1 |
| GO:0004872 | receptor activity (MF) | 96 | 7.74E-07 | 6.78E-05 | NR2C1,KLRG1,PAQR6,GABRB1,GRK5,PPARG,DBI,LRRN2,GRM7,CD244,IL22RA1,IFNGR1,NETO2,SORT1,TBXA2R,ADORA1,SSTR1,CD86,CD209,OR51Q1,LRP5,CRHR1,RXFP1,VAC14,OR6K2,CUBN,HAVCR1,GRIK3,ACVRL1,MC3R,OR10P1,CCR10,SCARB2,FZD9,CD180,TEK,LIFR,NGFR,PKHD1L1,ZFYVE9,FOLR1,NMBR,BDKRB2,IL20RA,RYR1,DAG1,ITGA5,CD36,RRH,CALCRL,NTRK2,PTPRA,NCOA3,GPR137B,ADRB1,GABRE,CIDEB,FGFRL1,SCARB1,GABRP,TNFRSF10A,MRC2,CHRNA10,ITGB4,KLRF1,CHRNA3,GRM5,ELTD1,HRH3,CRCP,TRPC4,FZD5,ERBB4,TIE1,CRLF3,NPR3,TAS2R10,GPR146,CCR7,CCR3,CXADR,NR2E1,OR2T5,TACR3,GCGR,NR2F2,DDR1,GPR116,IL28RA,P2RY2,TREM2,GPR45,RARG,BMPR1B,NPY1R,RARB |
| GO:0016491 | oxidoreductase activity (MF) | 38 | 1.10E-06 | 8.80E-05 | FADS2,HSD17B2,SQLE,SCCPDH,PGD,ALDH9A1,ALDH1A2,GPX3,DCT,CP,FASN,RDH11,FDFT1,AIFM3,LOXL3,F8,SCD,FADS1,ASPHD1,RETSAT,HTATIP2,QDPR,ALDH1L1,MPO,DHRS13,CRYM,DHCR7,ALDH3A2,FADS3,NDUFS1,PIPOX,PAOX,NOS3,ALDH4A1,HSD17B8,TM7SF2,ALDH2,FOXRED1 |
| GO:0030165 | PDZ domain binding (MF) | 14 | 2.25E-06 | 0.000167 | GRM7,GRASP,ARHGEF16,FRZB,FZD9,PLEKHA1,SNTB1,SFRP2,MPP3,ADRB1,FZD5,SLC9A3R1,CXADR,DLG3 |
| GO:0042813 | Wnt-activated receptor activity (MF) | 7 | 4.60E-06 | 0.000317 | LRP5,FRZB,FZD9,SFRP2,LRP5L,FZD5,TSPAN12 |
| GO:0016717 | oxidoreductase activity, acting on paired donors, with oxidation of a pair of donors resulting in the reduction of molecular oxygen to two molecules of water (MF) | 4 | 8.89E-06 | 0.000571 | FADS2,SCD,FADS1,FADS3 |
